# Formation of large viroplasms and virulence of *Cauliflower mosaic virus* in turnip plants depend on the N-terminal EKI sequence of viral protein TAV

**DOI:** 10.1371/journal.pone.0189062

**Published:** 2017-12-18

**Authors:** Angèle Geldreich, Gabrielle Haas, Julie Kubina, Clément Bouton, Mélanie Tanguy, Mathieu Erhardt, Mario Keller, Lyubov Ryabova, Maria Dimitrova

**Affiliations:** Institut de Biologie Moléculaire des Plantes, CNRS UPR2357, Université de Strasbourg, Strasbourg, France; University of Basel, SWITZERLAND

## Abstract

*Cauliflower mosaic virus* (CaMV) TAV protein (TransActivator/Viroplasmin) plays a pivotal role during the infection cycle since it activates translation reinitiation of viral polycistronic RNAs and suppresses RNA silencing. It is also the major component of cytoplasmic electron-dense inclusion bodies (EDIBs) called viroplasms that are particularly evident in cells infected by the virulent CaMV Cabb B-JI isolate. These EDIBs are considered as virion factories, vehicles for CaMV intracellular movement and reservoirs for CaMV transmission by aphids. In this study, focused on different TAV mutants *in vivo*, we demonstrate that three physically separated domains collectively participate to the formation of large EDIBs: the N-terminal EKI motif, a sequence of the MAV domain involved in translation reinitiation and a C-terminal region encompassing the zinc finger. Surprisingly, EKI mutant TAVm3, corresponding to a substitution of the EKI motif at amino acids 11–13 by three alanines (AAA), which completely abolished the formation of large viroplasms, was not lethal for CaMV but highly reduced its virulence without affecting the rate of systemic infection. Expression of TAVm3 in a viral context led to formation of small irregularly shaped inclusion bodies, mild symptoms and low levels of viral DNA and particles accumulation, despite the production of significant amounts of mature capsid proteins. Unexpectedly, for CaMV-TAVm3 the formation of viral P2-containing electron-light inclusion body (ELIB), which is essential for CaMV aphid transmission, was also altered, thus suggesting an indirect role of the EKI tripeptide in CaMV plant-to-plant propagation. This important functional contribution of the EKI motif in CaMV biology can explain the strict conservation of this motif in the TAV sequences of all CaMV isolates.

## Introduction

Virus-infected cells often contain cytoplasmic and/or nuclear inclusion bodies mainly composed of viral proteins and called viroplasms. Viroplasms are considered for many viruses as viral factories providing a physical scaffold to concentrate viral components and host factors within specific sites, and thereby increasing the efficiency of genome replication and/or assembly of viral particles [1]. In cells infected by some animal viruses, viroplasms result from the assembly of small aggregates that are transported by dynein along microtubules to the microtubule organization centre at the periphery of the nucleus, where they recruit cellular proteins and mitochondria [2–4]. These viroplasms resemble aggresomes that naturally occur in cells to reduce the toxicity of misfolded proteins and make them susceptible to proteolysis by the proteasome and/or by autophagy [2]. These inclusion bodies appear during the early steps of viral infection, dramatically alter the cell ultrastructure and differ from aggregates of overproduced viral proteins that accumulate late in infection. Most animal and plant RNA viruses modify membranes usually deriving from the secretory pathway, endoplasmic reticulum and Golgi apparatus, or from organelles such as mitochondria or peroxisomes, where they anchor their replication complexes and establish virus assembly sites [1]. RNA viruses belonging to the *Reoviridae* family also form viroplasms in which virions are produced, both in animals [5] and plants [6]. Viroplasms are also observed in cells infected by plant DNA viruses such as *Cauliflower mosaic virus* of the *Caulimoviridae* family [7–9].

*Cauliflower mosaic virus* (CaMV) is a plant pararetrovirus and the type member of the *Caulimovirus* genus [10,11]. Its circular double-stranded DNA genome (~8 kbp) is replicated through reverse transcription of the pre-genomic 35S RNA. The six proteins (P1 to P6) encoded by the CaMV genome are expressed from the polycistronic 35S RNA, whereas P6, also called TAV (TransActivator/Viroplasmin), is primarily synthesized from the monocistronic 19S RNA. TAV (62 kDa) is a nucleocytoplasmic shuttling protein essential for CaMV infectivity [9,12]. It is the major determinant of host specificity [13,14] and expression of symptoms [15,16] but it also triggers a hypersensitive response in *Nicotiana* plant species depending on the CaMV strains [17]. TAV interferes with plant defence mechanisms by inhibiting signalling responses to salicylic acid [18] and by suppressing RNA silencing possibly *via* the interaction with DRB4, the cofactor of DCL4 [12,19]. TAV also promotes the reinitiation of translation along the polycistronic 35S RNA thanks to its multiple interactions with the translation machinery [20], with a specific plant protein called RISP [21], and TOR protein kinase [22], thus enabling the synthesis of the full set of viral proteins [11,20]. In addition, TAV is required for an efficient replication of the CaMV genome, but the underlying mechanism is still unclear [23]. TAV might be involved in the assembly and the transport of CaMV particles, since it interacts with the capsid protein (P4), with CHUP1, a plant protein responsible for moving chloroplasts on actin microfilaments, and with plasmodesmal proteins [24–27]. Finally, TAV is also the major component of membrane-free amorphous electron-dense inclusion bodies/viroplasms (EDIBs) [8,9,28], easily distinguishable by electron microscopy in the cytoplasm of CaMV-infected cells from electron-lucent inclusion bodies/viroplasms (ELIBs), which are mainly constituted of P2 protein and are used as platforms for plant-to-plant transmission of CaMV by aphids [29]. EDIBs result from the self-association of TAV molecules without the assistance of any other viral protein [9,28]. Small TAV aggregates move along the actin filaments network, but they appear stationary on microtubules [26]. It has been proposed that EDIBs could be considered as mobile viral factories, which, during the late events of infection, dock and deliver virions to the tubules formed by movement protein P1 in plasmodesmata [26,30].

EDIBs used to be considered as containers of overexpressed non-functional TAV protein, until it was shown that they are the sites of CaMV DNA replication, virus assembly and storage of newly formed viral particles. The translation of CaMV transcripts is supposed to occur within or close to EDIBs, since the latter are surrounded by numerous ribosomes. Heterologous proteins (*i*.*e*. interferon) expressed from recombinant CaMV DNA have been detected in proximity of EDIBs [31]. These observations explain why it is generally accepted that EDIBs are CaMV factories and, consequently, why they are considered to be mandatory for CaMV infectious cycle.

CaMV-infected cells contain many EDIBs with areas ranging from 0.3 to 10 μm^2^, depending on both the virus strain and the host plant [32]. Virulent CaMV Cabb B-JI strain triggers in *Brassicaceae* the formation of unusually large perinuclear EDIBs (8 to 10 μm^2^) that arise from the gradual assembly of small TAV-aggregates (0.1 to 1 μm^2^) during the course of infection. On the contrary, other CaMV strains such as D/H or CM1841 only form small viroplasms at the final stage of infection [32]. The reason for these size differences is to date not fully understood. We have shown previously that the formation of large EDIBs is impaired in cultured tobacco BY2 cells [9] upon mutation of the EKI tripeptide (amino acids 11–13) of TAV, to AAA (mutant called TAVm3), and that this mutation did not abolish CaMV infectivity in *Arabidopsis thaliana* [12]. These observations suggested that large EDIBs might be dispensable for the virus. However, we could not totally exclude that EDIBs can still assemble in a viral context in host plants. Recently, it was shown that mutations within a 35 amino acids region (subdomain D3b) in the central part of TAV halved the size of EDIBs and CaMV infectivity [33].

Here we demonstrate that two other independent domains of TAV are required for the formation of EDIBs, in addition to the EKI motif and the recently described subdomain D3b [33]. We also show that the EKI mutation impairs the formation of large EDIBs in turnip plants, and thereby the virulence of the Cabb B-JI isolate, but does not affect CaMV systemic infection. Finally, we demonstrate that the absence of large EDIBs affects CaMV protein synthesis and capsid assembly, and dramatically reduces CaMV DNA replication efficiency and the subsequent production of infectious particles.

## Materials and methods

### Virus and host plants

All experiments were performed with the CaMV Cabb B-JI isolate. Three week-old turnip plants (*Brassica rapa*, cv. Tokyo) were mechanically inoculated with *Sal*I-linearized pMD324 or pGH plasmids coding for wild-type TAV or the mutated version TAVm3, respectively [12]. Inoculations were performed using, per plant, 10 μg of linearized CaMV DNA diluted in 20 μl sterile water. Ten turnip plants were inoculated with wild type or mutated viral DNA per assay. Infected leaves were harvested 21 days post-inoculation (dpi), ground in liquid nitrogen, and stored at -80°C. The results reported in this study were exclusively obtained from systemically infected tissues. Transgenic *Arabidopsis thaliana* (ecotype Columbia) expressing CaMV TAV or TAVm3 were described in [12].

### Plasmid constructions

The pCK-EGFP vector was used to construct the recombinant plasmids encoding the fusion proteins, consisting of enhanced green fluorescent protein (EGFP) and wild-type or mutant CaMV TAV. Full-length CaMV ORF VI and 3’ truncated ORF VI sequences were obtained by PCR performed on pETKaKS.6 recombinant plasmid containing the complete ORF VI of Cabb B-JI isolate [34] with appropriate primers. Amplification products were cloned in *Nco*I restriction site of the pCK-EGFP vector and verified by PCR to be in fusion and in frame with the 3’ end of the EGFP encoding sequence. Deletions and point mutations were introduced in the recombinant plasmid encoding EGFP:TAV by site-directed mutagenesis using specific primers, as previously described [9]. CaMV ORF VI sequence was also amplified using two primers carrying at their 5’ ends *Bsr*GI and *Xba*I sites, respectively, and cloned in these restriction sites of the pmRFP vector, in fusion with the sequence encoding the red fluorescent protein (mRFP). Forward and reverse primers used to generate ORF VI and its mutants are listed in S1 Table. Error-free recombinant plasmids were identified by DNA sequencing.

### Purification of CaMV particles

CaMV particles were extracted from infected turnip leaves ground in the presence of 1M urea to disrupt viroplasms and purified on a 10–40% sucrose gradient as previously described [35]. The concentration of purified virus was determined by spectrophotometry (one OD at 260 nm corresponding to 7 mg/ml of virions) [35].

### Analysis of CaMV proteins in infected turnip plants

Total proteins from systemically infected turnip leaves were extracted in Laemmli buffer 2X concentrated, containing 8 M urea, and fractionated by sodium dodecylsulphate-polyacrylamide gel electrophoresis (SDS-PAGE) 12%. After migration, proteins were electroblotted on PVDF membrane (Immobilon-P, Millipore) that was then stained with Coomassie blue in order to detect ribulose 1,5, bisphosphate carboxylase/oxygenase large subunit (RbcL) used as loading control. CaMV TAV, P4, P3, P2 and P1 proteins were detected using polyclonal antisera at a dilution of 1:10,000, alkaline phosphatase-conjugated goat anti-rabbit IgG antibodies (Biosys) at a dilution of 1:5,000 and the colorimetric substrate BCIP (5-Bromo- 4-Chloro-3-Indolyl Phosphate) / NBT (Nitroblue Tetrazolium) detection system (Promega)). Anti-P1 and anti-P2 antibodies were kindly provided by Andy Maule (John Innes Centre, Norwich, England) and Stéphane Blanc (INRA, Montpellier, France), respectively, and anti-P3 [36], anti-P4 [37] and anti-TAV [38] polyclonal antisera were previously obtained and tested in our laboratory. The amounts of viral proteins were quantified using ImageJ software (http://rsb.info.nih.gov/ij/) based on the intensity of the signal obtained for each of the 5 viral proteins relative to the RbcL control protein. The means for each viral protein from wild-type CaMV and CaMV-TAVm3-infected plants were compared using a Student’s 2-samples t-test and those for wild-type CaMV were set at 100%.

### 26S Proteasome inhibition in transgenic *Arabidopsis* seedlings

Eight-days-old transgenic *A*. *thaliana* seedlings, expressing CaMV TAV protein [12], grown on solid agar plates containing Murashige and Skoog (MS) medium, were carefully removed and washed in 10 mM MES (2-(N-morpholino)ethanesulfonic acid)) pH 5.7. Eighty seedlings were transferred into 7 mL of MES buffer containing 100 μM MG132 in dimethylsulphoxid (DMSO) (Selleckchem) or, for the mock treatment, DMSO only, infiltrated with these solutions under vacuum for 10 minutes and then incubated under gentle agitation at 21°C. Ten seedlings were collected at the indicated time points, frozen in liquid nitrogen and ground in a Precellys® homogenizer at 6,000 rpm for 7 seconds. Proteins of the crude extracts were quantified with amido black and finally, analysed by western blotting using rabbit polyclonal antibodies against CaMV TAV at a dilution of 1:10,000 and RGA DELLA (PhytoAB), provided by Patrick Achard (IBMP, Strasbourg, France), at a dilution of 1:1,000, secondary goat anti-rabbit antibodies conjugated to HRP (Horse Radish Peroxydase) (Thermo Fischer Scientific, and luminol-based enhanced chemiluminescence substrate (Lumi-Light^Plus^ Western Blotting Substrate, Roche).

### Analysis of viral DNA from CaMV-infected plants by semi-quantitative (RT)-PCR

At 21 dpi, two discs (diameter 0.5 cm) sampled from CaMV or CaMV-TAVm3-infected turnip leaves were ground in liquid nitrogen in a Precellys® homogenizer at 6,000 rpm twice for 20 seconds. After DNA denaturation in 200 μL 0.5 N NaOH per plant crude extract, 5 μL were added to 45 μL 0.1 M Tris-HCl pH 8 for neutralization. PCRs were performed on 3 μL of the treated crude extract with couples of specific oligonucleotides to amplify sequences from CaMV DNA and the actin gene *act2* used as an internal control (S1 Table). PCRs were run on a T gradient thermocycler Biometra using the Go Taq®flexi DNA polymerase (Promega). The PCR products obtained after 22 to 30 cycles of amplification were analysed on a 1% agarose gel and revealed by ethidium bromide staining.

### Detection of CaMV 19S and 35S RNAs, and virus-derived small RNAs

Total RNA from infected plant leaves was extracted using the RNeasy plant mini kit (Qiagen) and followed by a DNase I (Promega) treatment to eliminate residual DNA. RNA was precipitated with isopropanol and dissolved in 50% formamide. Northern blot analyses of low and high molecular weight RNA were performed with 10 and 5 μg of total RNA, respectively, as previously described [12]. CaMV 35S and 19S RNAs were detected using as probes DNA oligonucleotides end-labelled with [γ-^32^P] ATP (3,000 Ci/mmole) complementary to ORF VI and ORF II, respectively. Virus-derived small RNAs (vsRNAs) were detected with [α-^32^P] dCTP (800 Ci/mmole) radiolabelled probes resulting from random priming reactions on pMD324, which contains the CaMV full-length genome [12].

### Biolistic experiments

CaMV TAV protein and the mutated versions fused to EGFP, and TAV fused to mRFP, were transiently expressed in BY-2 tobacco suspension cells (*Nicotiana tabacum* cv Bright Yellow 2). Cells were subcultured weekly and harvested 3 days after medium renewal for biolistic transfection. Cells were filtered onto Whatman disks. Particles preparation and bombardment assays were performed as follows: 2 mg of 1.1 μm tungsten particles (Bio-Rad, Hercules, CA) were immersed in 1 mL of absolute ethanol for 20 min. Dried particles were then successively mixed with 10 μg of recombinant plasmid DNA (pCK-EGFP-TAV or derivatives, or pmRFP-TAV) supplemented with 18% glycerol, 750 mM CaCl_2_, and 90 mM spermidine in a final volume of 30 μL. The firing distance was 11 cm and the helium pressure 7 bars. After bombardment, cells were transferred to 0.8% agar MS media plates and incubated in the dark at 28°C. Transfected BY-2 cells were collected under HBO binoculars (excitation/emission wavelength 488/505 to 545 nm) 20 h after bombardment and before further treatment and/or observations with a Zeiss LSM700 confocal microscope (Jena, Germany).

### Immunofluorescence analysis

Tobacco BY-2 cells, transfected with constructions encoding TAV and its mutated versions fused to EGFP or mRFP, were observed between slide and cover slip with laser scanning confocal microscope (LSCM). EGFP and mRFP were detected after excitation at 488 nm and 568 nm with argon and HeNe laser, respectively, and using an appropriate emission filter to collect the signals from the optical section. Cells were observed 16 h and 24 h after bombardment.

For immunofluorescence localization studies, protoplasts were prepared from CaMV-infected turnip plants as described in [39] and fixed for 15 min under gentle shaking in protoplast medium containing 1% glutaraldehyde. Thereafter, they were washed three times with the protoplast medium, once with the medium diluted volume to volume with phosphate-buffered saline (PBS) and then again with PBS. A sample of protoplasts was mounted on poly-L-Lys–coated cover slip, allowed to settle for 1 h at room temperature, and then treated overnight at 4°C in a 0.1% sodium borohydride solution. Protoplasts were incubated for 1 h in a blocking solution (5% acetylated bovine serum albumin [BSAc, Aurion, Wageningen, The Netherlands]), 5% normal goat serum, and 0.1% cold water fish skin gelatine prepared in PBS) and then overnight, with polyclonal anti-TAV, anti-P4 or anti-P2 antibodies, at a dilution of 1:500. After six washes with PBS containing 0.1% BSAc, protoplasts were treated with goat or mouse anti-rabbit IgG coupled to Alexa 488 or Alexa 568 respectively (Molecular Probes, Eugene, OR) at a dilution of 1:300 for 12 h. After several washes in PBS containing 0.1% BSAc, protoplasts were examined by LSCM.

### Electron microscopy of CaMV-infected turnip cells

Infected leaves, cut into even strips (0.1 x 1 cm), were fixed overnight in 4% glutaraldehyde with 10% picric acid, then successively stained with 2% uranyl acetate for 2 h each, and with 0.1% (v/v) osmium tetroxide in 150 mM phosphate buffer pH 7.2. Samples were dehydrated through an ethanol series and infiltrated with EPON812 medium-grade resin (Polysciences). Polymerization was performed for 72 h at 60°C. Ultrathin sections (90 nm) were cut using an ultracut E microtom (Reichert) and collected on grids coated with formvar (Electron Microscopy Sciences). Finally, samples were visualized with a Hitachi H-600 transmission electron microscope operating at 75 kV and viroplasms surface was measured with ImageJ software.

## Results

### Formation of large electron-dense inclusion bodies (EDIBs) involves three physically independent regions of TAV

**Fig 1:**
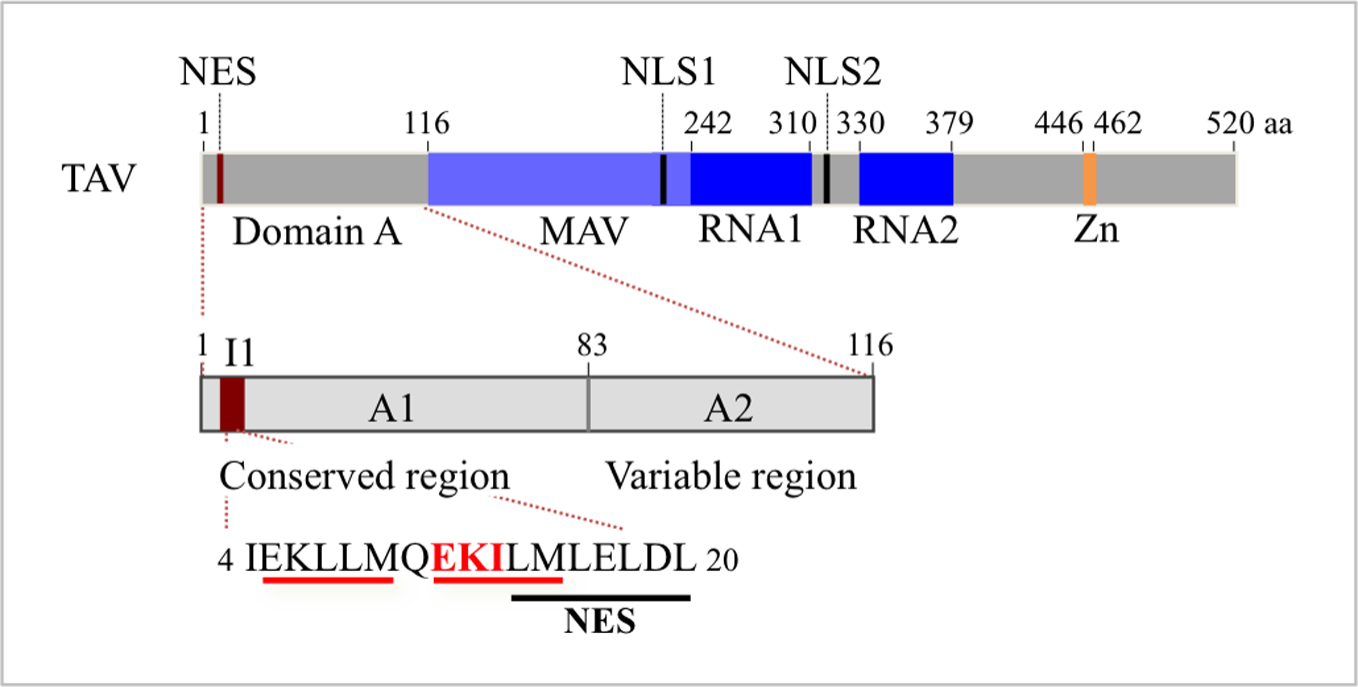
Schematic representation of CaMV TAV protein functional domains. Numbers above the boxes indicate the amino acids and delimitate the functional domains of TAV: domain A (grey box, amino acids 1–116); MAV (light blue box, amino acids 117–242); RNA1 (dark blue box, amino acids 243–310); RNA2 domain (second dark blue box, amino acids 330–379), and the zinc finger (orange vertical bar, amino acids 446–462). Positions of the single NES and the two NLSs are indicated with red and black vertical bars, respectively [9,12]. Below is detailed domain A with its subdomains, the conserved A1 region (amino acids 1–83) and the variable A2 region (amino acids 84–116) [9]. The invariant sequence I1 is represented with a dark red box and its sequence is given with the EKI motif in red letters; the motif is substituted by three alanines (AAA) in TAVm3 mutant. The tandem repeated sequence EKI/LLM and the NES are underlined in red and black, respectively.

Several functional domains of TAV were mapped (Fig 1): domain A, divided into A1 and A2 subdomains, and involved in host specificity, symptomatology and self-assembly; MAV (MiniTAV) domain and RNA binding domain 1 (RNA1), also known as MBD (Multiple Binding Domain), are both required for reinitiation of translation and bind double-stranded RNA and RNA-DNA hybrids, respectively; RNA binding domain 2 (RNA2) which binds single-stranded RNA, and, finally, a zinc finger (Zn) domain. Previous studies performed in cultured tobacco BY-2 cells, non-host for CaMV, indicated that the EKI tripeptide located in TAV subdomain A1 of the Cabb B-JI isolate plays an essential role in the formation of EDIBs [9].

**Fig 2:**
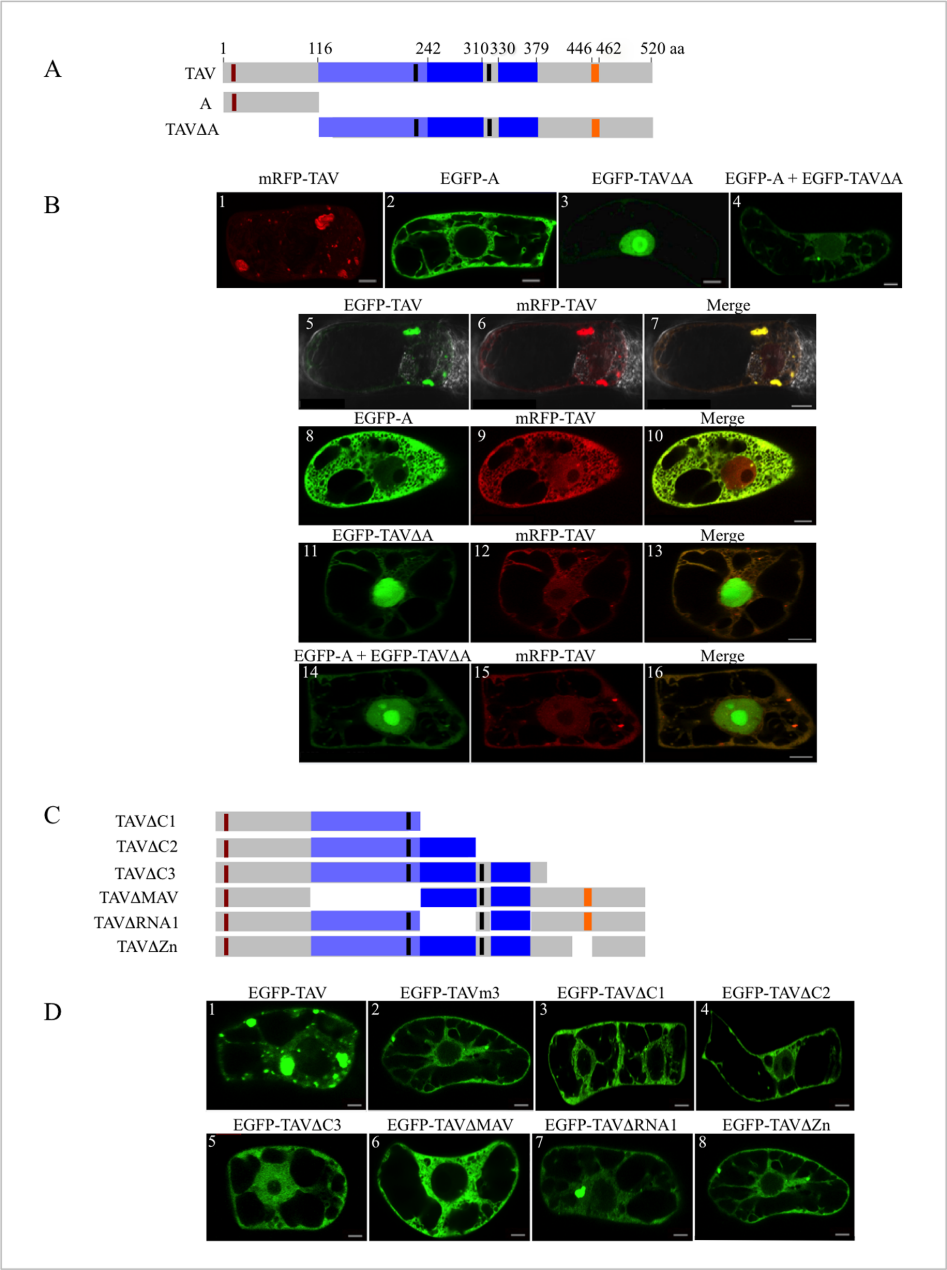
Characterization of CaMV TAV regions involved in the formation of inclusion bodies. (A, C) Schematic representation of TAV and TAV deletion mutants: A, TAVΔA, TAVΔC1, TAVΔC2, TAVΔC3, TAVΔMAV, TAVΔRNA1 and TAVΔZn. Numbers above the diagram of full-length TAV indicate the amino acids. The functional domains of TAV are depicted as in Fig 1. (B) Transient expression of mRFP-TAV (panel 1), EGFP-A (panel 2), EGFP-TAVΔA (panel 3), and EGFP-A and EGFP-TAVΔA together (panel 4) in tobacco BY-2 cells, and competition assays performed in BY-2 cells co-transfected with plasmids encoding mRFP-TAV and EGFP-TAV (panels 5–7), EGFP-A (panels 8–10), EGFP-TAVΔA (panels 11–13) and both EGFP-A and EGFP-TAVΔA (panels 14–16), respectively. (D) Identification of TAV domains involved in the formation of inclusion bodies by transient expression of EGFP-TAV deletion mutants in BY-2 cells. Observations of TAV and TAV mutants fused to EGFP (B, D) or mRFP (B) were made 16 h after transfection, by LSCM. The LSCM settings and acquisition conditions of the images (single sections) were identical in all (B) and (D) panels. Scale bars: 10 μm.

To further investigate the mechanism involved in viroplasm formation, we co-transfected tobacco BY-2 cells with recombinant plasmids encoding full-length TAV fused at its N-terminus to mRFP (mRFP-TAV), and two truncated TAV proteins, fused at their N-termini to EGFP (EGFP-A and EGFP-TAVΔA), to verify by competition assays if, in addition to domain A, other TAV sequences are involved in the formation of EDIBs (Fig 2A and 2B). Having already shown in a previous study that the fusion of EGFP did not modify the capacity of TAV to form EDIBs in BY-2 cells [9], we observed the cells using LSCM, 16 h and 24 h after transfection. mRFP-TAV protein assembled into large IBs when expressed alone (Fig 2B, panel 1), thus demonstrating that, as for EGFP, the fusion of mRFP at the N-terminus of TAV did not hinder TAV self-association. When mRFP-TAV was co-expressed with EGFP-TAV in BY-2 cells, the two TAV fusion proteins co-localized, as evidenced by the yellow fluorescent foci observed in the merge image (Fig 2B, panels 5–7). As previously reported [9], EGFP-A diffused in the cytoplasm (Fig 2B, panel 2) and EGFP-TAVΔA was retained in the nucleus (Fig 2B, panel 3). Their cellular localization is due to the presence of a NES (in domain A) and two NLSs (in the downstream region) that are involved in TAV nucleo-cytoplasmic shuttling (Fig 1). When mRFP-TAV was co-expressed in tobacco cells with EGFP-A (Fig 2B, panels 8 to 10; S1A Fig) or EGFP-TAVΔA (Fig 2B, panels 11–13; S1B Fig) or both (Fig 2B, panels 14–16; S1C Fig), it did not form any large IBs but diffused essentially in the cytoplasm, despite the nuclear accumulation of EGFP-TAVΔA. Few fluorescent foci were detected in some BY2-cells (Fig 2B, panels 11–16; S1B and S1C Fig) indicating that EGFP-A and/or EGFP-TAVΔA did not totally prevent mRFP-TAV self-assembly. The latter was probably overexpressed in these cells compared to EGFP-TAV deletion mutants, as evidenced by the weak green fluorescence of the foci (Fig 2B, panels 11 and 14; panels 1 in S1B and S1C Fig) and their orange colour in the merge images (Fig 2B, panels 13 and 16; panels 4 in S1B and S1C Fig). The capacity of EGFP-TAVΔA to interfere with mRFP-TAV self-association was surprising, since TAVΔA did not bind full-length TAV *in vitro*, in far western assays, contrary to domain A [9]. No TAV bodies were formed even when EGFP-A and EGFP-TAVΔA were expressed together in BY-2 cells in order to reconstitute *in trans* a complete TAV protein (Fig 2B, panel 4). Taken together, these results indicate that both TAV mutants out-competed the interactions between full-length TAV molecules, thus impairing the formation of EDIBs. This reinforced our hypothesis that one or several sequences of TAV, located downstream of domain A, are required for an efficient formation of IBs.

To identify these sequences, we analysed in tobacco BY-2 cells, the behaviour of EGFP-fused TAV mutants with C-terminal deletions (TAVΔC1: residues 1–242, TAVΔC2: residues 1–313 and TAVΔC3: residues 1–400) or functional domain deletions (TAVΔMAV, TAVΔRNA1, TAVΔRNA2 and TAVΔZn) (Fig 1, Fig 2C and 2D). For comparison, we also ectopically expressed EGFP-TAV (Fig 2D, panel 1) and EGFP-TAVm3 (Fig 2D, panel 2) in BY-2 cells. None of the C-terminally truncated TAV mutants that all possess the domain A generated inclusion bodies (Fig 2D, panels 3–5), confirming that additional domains are required for TAV self-assembly. The partial localization of EGFP-TAVΔC3 in the nucleoplasm, despite possessing both nuclear import and export signals, may be due to an unusual conformation of this TAV mutant, hiding the NES. Two regions of TAV involved in the formation of inclusion bodies could be unambiguously identified when we tested internally deleted TAV mutants in tobacco BY-2 cells: the MAV domain, where the relevant sequence could be restricted to positions 218 to 242, and the zinc finger-encompassing domain (positions 413–462). Their deletion abolished the formation of TAV bodies as evidenced by the diffused green fluorescence in the cytoplasm (Fig 2D, panels 6 and 8), whereas the deletion of RNA1 binding domain did not (Fig 2D, panel 7). The behaviour of EGFP-TAVΔRNA2 was uncertain since it formed small aggregates or showed a diffuse cytoplasmic localization depending on the assay (data not shown).

In conclusion, our results indicate that, in addition to the EKI motif, two other physically separated regions of TAV, MAV and the domain containing the zinc finger, are required for EDIB formation. They likely act collectively, since the deletion of each of these sequences is sufficient to impair this process.

### TAVm3 is unable to form large EDIBs in turnip plants in a viral or non-viral context

In previous studies, we noticed that the CaMV Cabb B-JI isolate encoding TAVm3 is as infectious in *A*. *thaliana* (ecotype Columbia) [12] as in turnip plants (unpublished data), suggesting that either TAVm3 forms EDIBs in a viral context or, on the contrary, that EDIBs are dispensable for CaMV infection.

**Fig 3:**
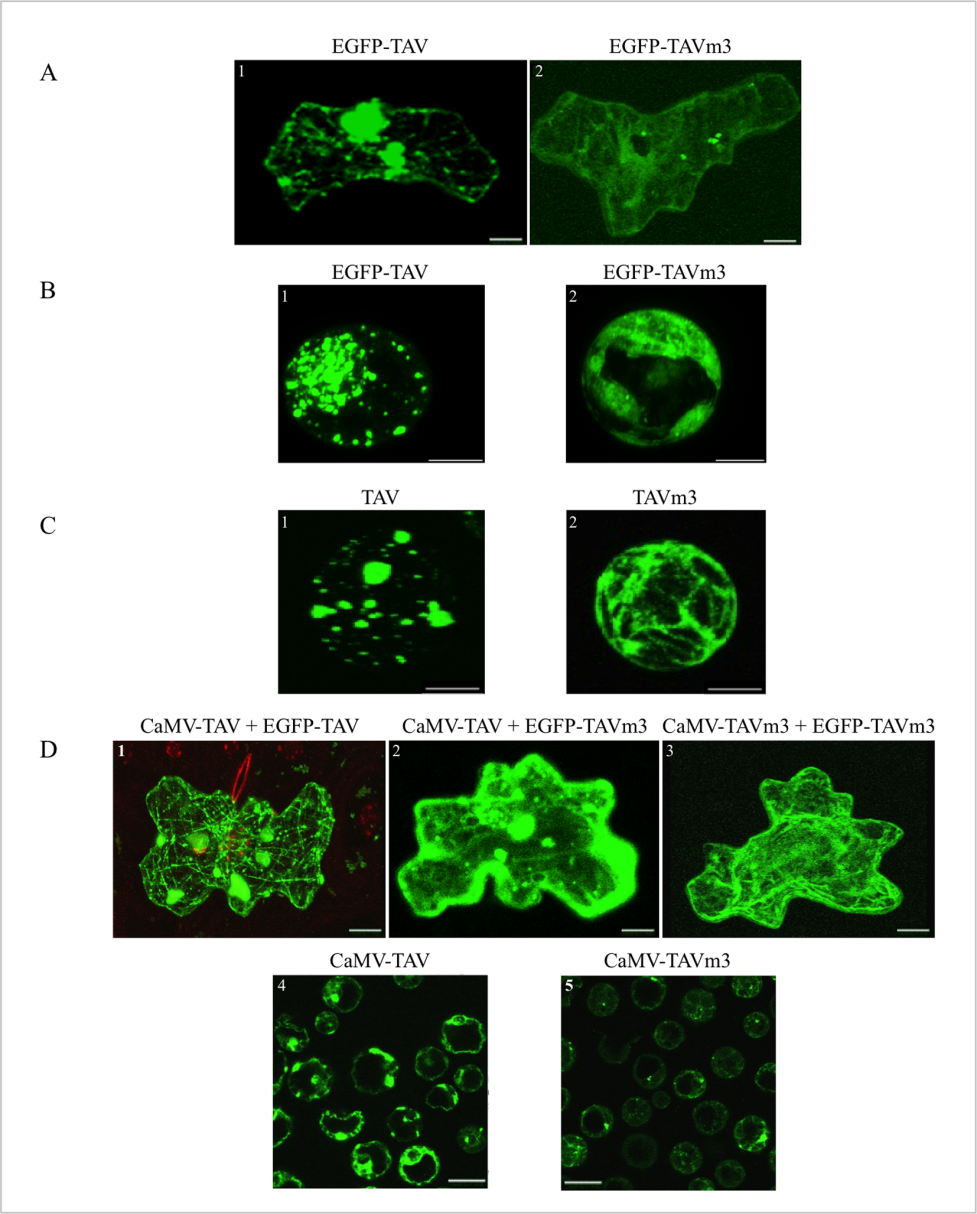
CaMV TAV and TAVm3 proteins expressed in turnip plants and *A*. *thaliana* in a viral or non-viral context. (A and B) Transient expression of EGFP-TAV and EGFP-TAVm3 in turnip epidermal cells bombarded with the corresponding pCK-EGFP recombinant plasmids (A, panels 1 and 2) and in *A*. *thaliana* (ecotype Columbia) protoplasts transfected by PEG with same constructions (B, panels 1 and 2). Cells were observed 16 h after transfection, by LSCM. (C) Immunolocalisation of TAV and TAVm3 (panels 1 and 2) in protoplasts obtained from transgenic *A*. *thaliana* (ecotype Columbia) lines expressing wild-type or TAVm3 [12]. Protoplasts were incubated with polyclonal anti-TAV antibodies [38] and with anti-rabbit IgG secondary antibodies conjugated to green fluorochrome Alexa 488. (D) Detection of TAV proteins by EGFP fluorescence, 21 dpi, in turnip epidermal cells from systemic leaves infected with wild-type CaMV (panels 1 and 2) or CaMV-TAVm3 (panel 3) through interactions with ectopic EGFP-TAV (panel 1) and EGFP-TAVm3 (panels 2 and 3) expressed upon bombardment with the corresponding plasmids. Immunofluorescence detection of TAV and TAVm3 in protoplasts isolated from CaMV-TAV and CaMV-TAVm3-infected turnip leaves (panels 4 and 5). The LSCM settings and acquisition conditions of the images were identical in all panels. Images in (A), (B), (C) and (D) (panels 1–3) are projections; those in (D) (panels 4–5) show single sections. Scale bars (A and D): 10 μm, (B and C): 5 μm.

To verify these hypotheses, we first expressed EGFP-TAV and EGFP-TAVm3 in turnip and in *A*. *thaliana* (ecotype Columbia) in a non-viral context. As in tobacco BY-2 cells (Fig 2), epidermal cells from turnip leaves bombarded with the plasmid coding for EGFP-TAV displayed green fluorescent aggregates of different sizes, among which were large TAV inclusion bodies (8–10 μm^2^) (Fig 3A, panel 1). On the other hand, EGFP-TAVm3 showed a diffused distribution in the cytosol of turnip cells transfected with the corresponding recombinant plasmid (Fig 3A, panel 2). Small fluorescent aggregates (< 1 μm^2^) were visible in some turnip cells, albeit to a lesser extent than with EGFP-TAV. In transient expression assays performed on *A*. *thaliana* protoplasts, EGFP-TAV and EGFP-TAVm3 fusion proteins both accumulated at the same levels (S2 Fig) and showed the same behaviour as in turnip and tobacco BY-2 cells (Fig 3B, panels 1 and 2) thus suggesting that TAV’s EKI mutation (and not a lower accumulation of EGFP-TAVm3) was responsible for the absence of large EDIBs. To also ensure that TAV and TAVm3 aggregation capacities were not influenced by their fusion to EGFP in these plants, we took advantage of transgenic *A*. *thaliana* lines expressing TAV and TAVm3 at comparable levels [12]. Immunofluorescence experiments performed on protoplasts isolated from these transgenic plants revealed that TAV and TAVm3 behaved identically as when they were fused to EGFP (Fig 3C, panels 1 and 2), thus demonstrating that the inability of TAVm3 to self-assemble into large EDIBs was related to the EKI mutation *per se*.

Next, turnip plants were mechanically inoculated with linear CaMV DNA, obtained by *Sal*I digestion of viral vectors [12], and encoding wild-type TAV (CaMV-TAV) or TAVm3 (CaMV-TAVm3), in order to determine the behaviour of TAV proteins in a viral context. Systemically infected turnip leaves were analysed to ensure that most of the inclusions were fully developed [32]. CaMV-TAV and CaMV-TAVm3-infected leaves were bombarded with plasmids encoding EGFP-TAVm3 or EGFP-TAV used as control, to detect TAV proteins produced in the course of CaMV replication cycle through interactions with their fluorescent counterpart (Fig 3D, panels 1–3). Ectopic EGFP-TAVm3 revealed in epidermal turnip cells infected with wild-type CaMV that TAV formed numerous cytosolic aggregates, highly variable in size (1–10 μm^2^), which were either free or appeared to be associated with the cytoskeleton (Fig 3D, panel 2), as reported in a previous study [26]. The fluorescent aggregates resulted from the interaction between EGFP-TAVm3 and TAV inclusions formed during the viral cycle, since EGFP-TAVm3 alone is unable to self-assemble in turnip cells (Fig 3A, panel 2). Ectopic EGFP-TAV in turnip cells infected with wild-type CaMV labelled large cytoplasmic aggregates and decorated the cytoskeleton network (Fig 3D, panel 1). On the other hand, EGFP-TAVm3 diffused in the cytoplasm or revealed small aggregates (< 1 μm^2^) (Fig 3D, panel 3), indicating that TAVm3 was unable, as in a non-viral context, to self-assemble into large IBs during an authentic viral infection. The observation of a population of protoplasts isolated from CaMV-TAVm3-infected systemic turnip leaves (Fig 3D, panel 5) and treated with anti-TAV antibodies and secondary antibodies coupled to Alexa 488, revealed that in fact only small cytosolic TAVm3 inclusion bodies were present in protoplasts, often along the plasma membrane, while those infected with wild-type CaMV systematically contained large EDIBs (Fig 3D, panel 4). Sequence analysis showed that CaMV progeny DNA maintained the EKI mutation in the tested plants after several serial passages of CaMV-TAVm3, indicating that EKI is not essential for CaMV infectivity, while the opposite was predicted since it is highly conserved among all CaMV isolates.

Taken together, our results show that the TAVm3 mutant, in *Brassicaceae*, is also unable to self-assemble into large IBs in a viral context, strongly suggesting that large EDIBs are dispensable for systemic CaMV infection of host plants.

### TAV’s EKI motif is important for CaMV Cabb B-JI virulence on turnip plants

The finding that the CaMV-TAVm3 mutant systemically infected *Brassicaceae* prompted us to investigate, in turnip, the biological relevance of EKI for CaMV pathogenesis.

**Fig 4:**
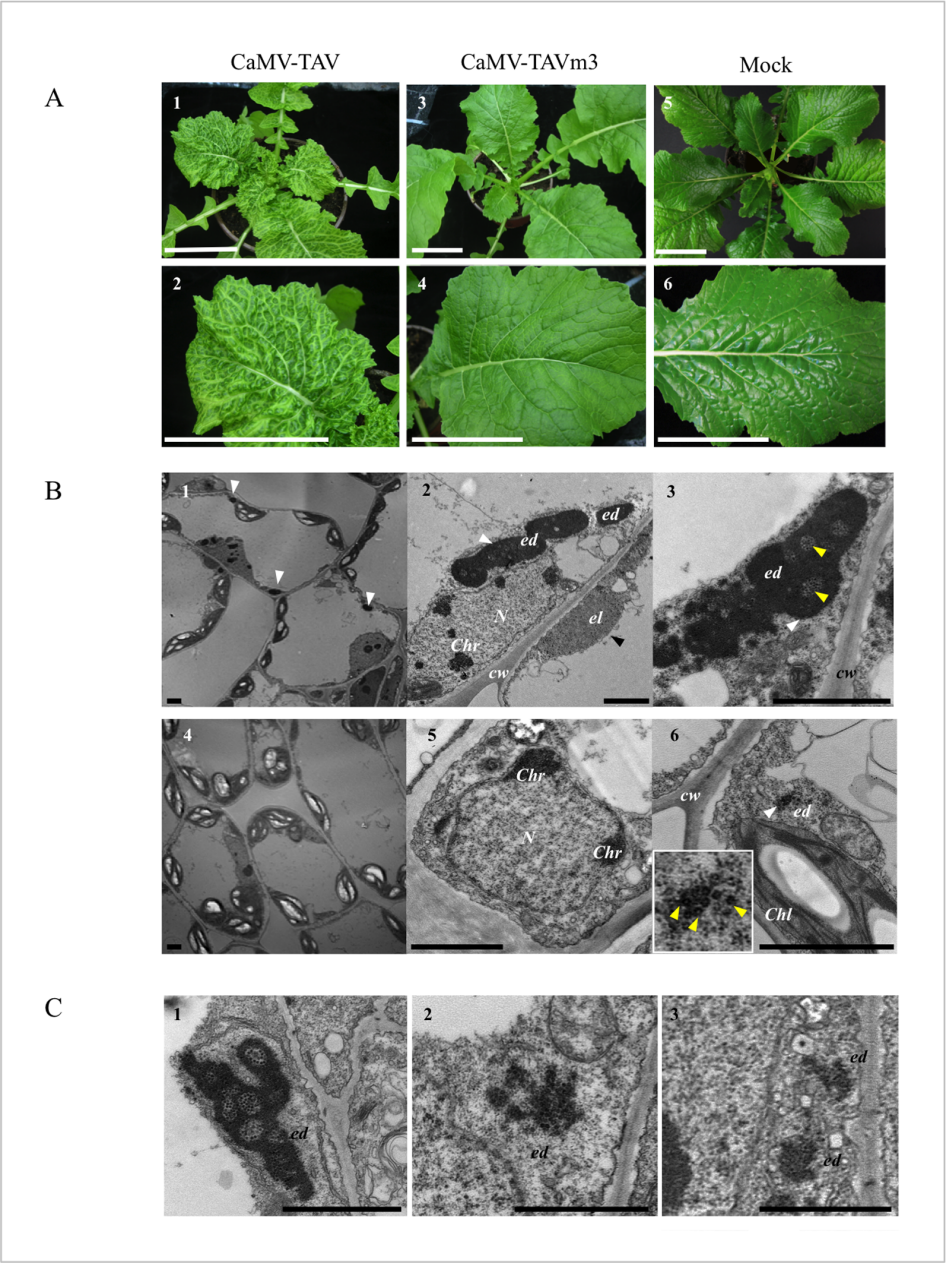
Phenotype of turnip and *A*. *thaliana* plants infected with wild-type CaMV or CaMV-TAVm3 mutant. (A) Symptoms expressed by turnip plants and systemic leaves infected with CaMV-TAV (panels 1 and 2) or CaMV-TAVm3 (panels 3 and 4) 21 dpi. A mock-inoculated turnip plant and leaf are shown (panels 5 and 6). Scale bars: 6 cm. (B) Photographs of turnip cells infected with CaMV (panels 1–3) or CaMV-TAVm3 (panels 4–6) observed by electron microscopy. Electron-dense viroplasms/aggregates (**ed**) and electron-lucent viroplasms (**el**) are pointed by white and black arrowheads, respectively. Panel 6: enlargement of the electron-dense body (**ed**). Yellow arrowheads indicate some CaMV particles. (C) Electron microscopy photographs of *Arabidopsis* cells from wild-type CaMV-infected plants (panel 1), CaMV-TAVm3-infected plants (panel 2) and a transgenic line expressing TAVm3 (panel 3). **Chl**: chloroplast; **Chr**: chromatin; **cw**: cell wall; **N**: nucleus. Scale bars (B-C): 2 μm.

Turnip plants inoculated with linearized CaMV-TAVm3 DNA were systemically infected 21 dpi, without any delay compared to plants inoculated with wild-type CaMV DNA. Sequence analysis of ORF VI after several rounds of virus replication or successive inoculations (> 10) of host plants with an infectious sap, showed that CaMV did not restore the EKI motif. Turnip plants infected with wild-type CaMV displayed severe symptoms (mosaic, vein clearing and stunting of plant and leaves) (Fig 4A, panels 1 and 2) whereas CaMV-TAVm3-infected plants showed very mild symptoms (faint chlorosis, no plant stunting) (Fig 4A, panels 3 and 4) and were thus hardly distinguishable from mock-inoculated plants (Fig 4A, panels 5 and 6). CaMV presence in systemic turnip leaves was confirmed by detection of viral DNA by PCR and viral proteins by western blot (data not shown). Disease symptoms became no more pronounced over time with CaMV-TAVm3, in contrast to those elicited by the wild-type virus.

**Fig 5:**
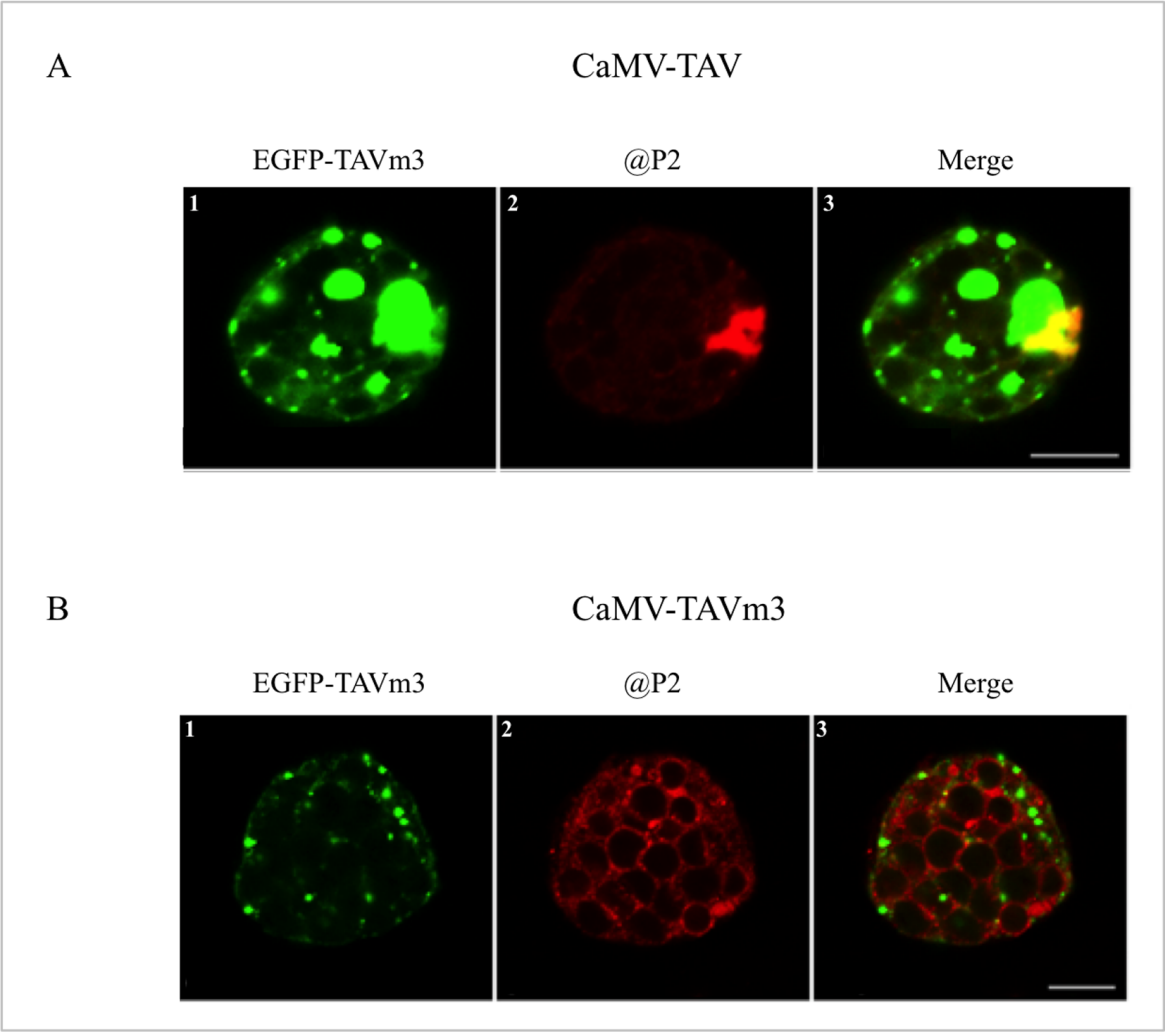
Immunolocalisation of electron-lucent inclusion bodies in protoplasts prepared from turnip plants systemically infected with wild-type CaMV or CaMV-TAVm3. Electron-dense inclusion bodies (EDIBs) were visualized in protoplasts infected with wild-type CaMV (A) or CaMV-TAVm3 (B) by transient expression of EGFP-TAVm3 (A, panel 1 and B, panel 1). Electron-lucent inclusion body/aggregates (ELIB) generated by CaMV P2 protein were observed by LSCM, after treatment of the infected and transfected protoplasts with polyclonal anti-P2 protein and secondary Alexa 568-conjugated antibodies (A, panel 2 and B, panel 2). Merge images showing the cellular distribution of EDIBs and ELIB, and TAVm3 and P2 bodies are presented in A, panel 3, and B, panel 3. All panels show single sections. Scale bars: 5 μm.

Electron-microscope observations of cellular ultrastructure were made on CaMV-infected systemic turnip leaves exhibiting a disease phenotype, to precisely assess the morphology of TAVm3-bodies. Photographs encompassing approximately 300 cells from four independent infection assays, were taken, all showing very similar results. Fig 4B shows 6 typical representative images. Large perinuclear EDIBs with vacuolar-like areas full of virions and a single ELIB, formed by P2 [40], were found in turnip cells infected with wild-type CaMV (Fig 4B, panels 1–3). Cells from turnip plants infected with CaMV-TAVm3 never contained such EDIBs, but in some cells we observed electron-dense areas certainly corresponding to the small inclusion bodies observed by LSCM (Fig 4B, panels 4–6). They also contained much less virions than wild-type CaMV-infected cells. Magnification of the electron-dense areas in CaMV-TAVm3-infected cells showed that they are irregularly shaped and granular compared to authentic EDIBs, suggesting that they may result from clumping of virus particles. Morphologically similar TAVm3 bodies were also observed in Arabidopsis plants infected with CaMV-TAVm3, as well as in transgenic Arabidopsis lines expressing TAVm3 (Fig 4C). Unexpectedly, CaMV-TAVm3-infected plants did not contain any ELIB, while this type of IB is always formed in CaMV-infected cells. To confirm this, protoplasts from turnip plants infected with CaMV or CaMV-TAVm3, were analysed by EGFP autofluorescence and anti-P2 immunofluorescence to detect both EDIBs and ELIB. CaMV-TAV- or CaMV-TAVm3-infected protoplasts were first transfected with the plasmid encoding EGFP-TAVm3 to reveal EDIBs (Fig 5A, panel 1, and 5B, panel 1). Then, protoplasts were fixed and treated with anti-P2 antibodies and Alexa 568-coupled secondary antibodies to detect ELIBs (Fig 5A, panel 2, and 5B, panel 2). Large EDIBs and a single large ELIB were clearly visible in protoplasts infected with wild-type CaMV (Fig 5A, panel 3; S3 Fig). By contrast, the P2 protein was dispersed throughout the cell, forming small aggregates (< 1 μm^2^) physically separated from those generated by TAVm3 (Fig 5B, panel 3; S4 Fig), thus confirming that ELIB was not formed when the EKI motif was mutated, despite the fact that P2 was expressed at a significant level (see below), and suggesting that native TAV and/or EDIBs are mandatory for the formation of the ELIB which serves as platform for aphid-transmission of CaMV [29].

In conclusion, mutating EKI dramatically affects the virulence of the CaMV Cabb B-JI isolate on turnip plants, as evidenced by (i) the mild phenotype exhibited by infected plants, and (ii) the few CaMV particles produced in the course of infection. Neither large EDIBs nor ELIB were formed in turnip plants infected with the CaMV-TAVm3 mutant.

### Large EDIBs are dispensable for CaMV infectivity but enhance the production of virus progeny

**Fig 6:**
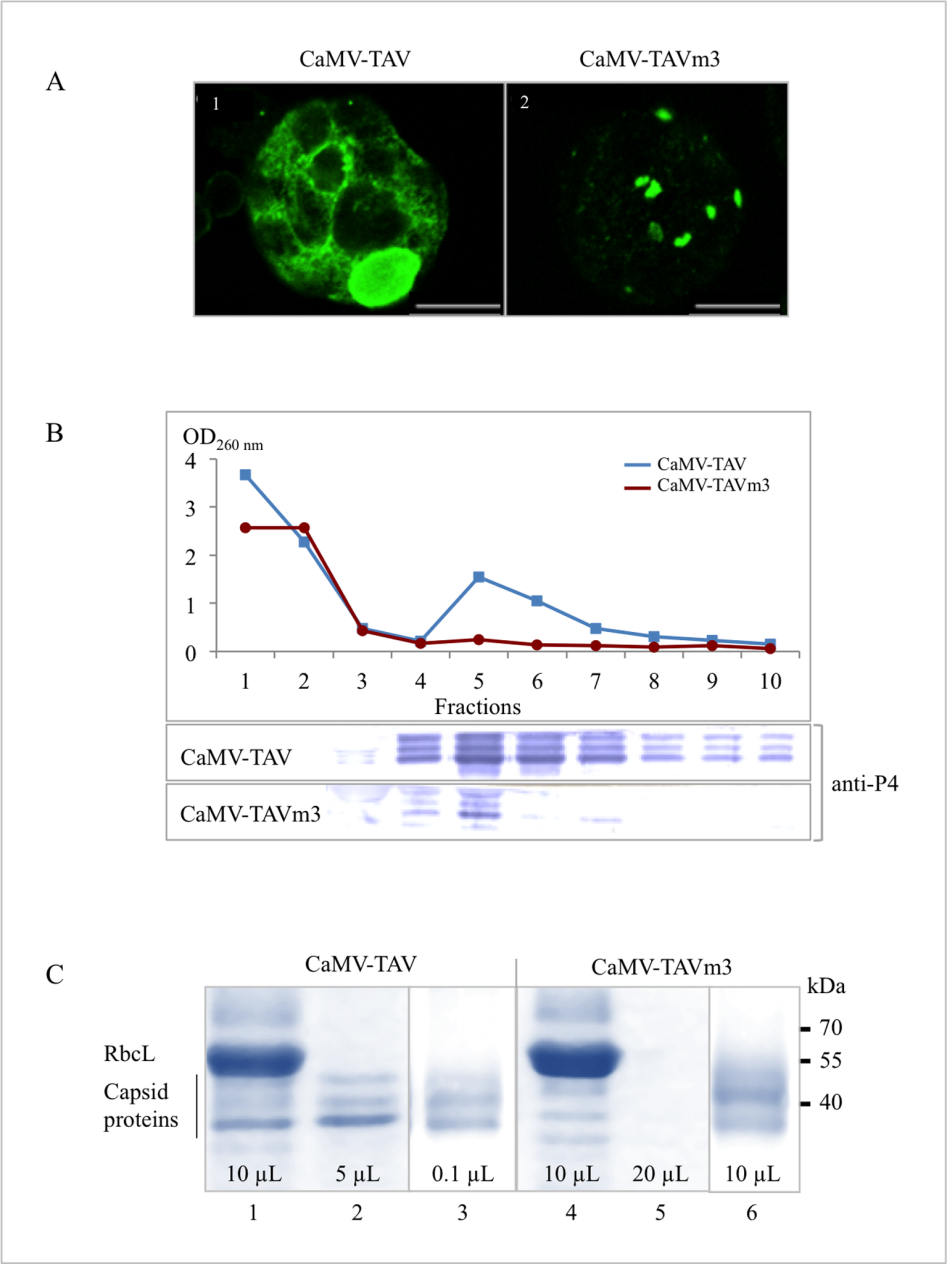
Purification of wild-type CaMV and CaMV-TAVm3 particles from infected turnip plants. (A) Immunolocalisation of CaMV and CaMV-TAVm3 capsids in protoplasts isolated from infected plants using polyclonal anti-P4 (pre-CP) antibodies and Alexa 488-coupled secondary IgG. Protoplasts were observed by LSCM. Images show single sections. (B) Purification of CaMV particles on sucrose density gradient: wild-type CaMV (blue line and squares) and CaMV-TAVm3 (red line and circles) were detected by measurement of OD_260nm_ of each 500 μL-fraction. Fractions 3–10 were tested for the presence of capsid proteins by immunodetection using anti-P4 antibodies. Peak fractions 4–8 corresponding to purified CaMV or CaMV-TAVm3, were pooled, concentrated and analyzed. (C) Analysis of CaMV-TAV (lanes 1 to 3) and CaMV-TAVm3 (lanes 4 to 6) particles (purified simultaneously and under strictly identical conditions) by 12% SDS-PAGE fractionation. Proteins in crude extracts before the gradient purification (aliquots of 10 μL) were revealed by Coomassie blue staining (lanes 1 and 4). Purified viruses from peak fraction 5 of the gradient (aliquots of 5 μL and 20 μL, respectively) were also analyzed by staining (lanes 2 and 5). Purified viruses from the pooled and concentrated peak fractions (aliquots of 0.1 μL and 10 μL, respectively) were immunodetected (lanes 3 and 6) using anti-P4 antibodies. RbcL and CaMV capsid proteins, and molecular markers are indicated at the left and right of panel C, respectively.

Electron microscopy observations revealed that turnip plants infected with CaMV-TAVm3 contained less virus particles than those infected with wild-type CaMV. Most CaMV particles in plants infected with CaMV-TAVm3 were essentially clustered in electron-dense areas, while virions were concentrated in the matrix of EDIBs in plants infected with wild-type CaMV (Fig 4); it was estimated that EDIBs contain 95% of the virus particles [41]. We could also indirectly confirm this observation by immunodetection of P4, the capsid proteins precursor (pre-CP), since wild-type CaMV-infected protoplasts displayed a cytosolic green fluorescence superimposed by a large green oval-shaped area corresponding to a perinuclear EDIB (Fig 6A, panel 1) while protoplasts infected with CaMV-TAVm3 exhibited fluorescence restricted to small areas (Fig 6A, panel 2). To definitely confirm that CaMV-TAVm3 mutant was produced in lower amounts than wild-type CaMV, virus particles from infected turnip plants 21 dpi were purified on a sucrose density gradient and quantified by spectrophotometry as shown in Fig 6B. Similar amounts of infected plant crude extracts, evidenced by the detection of the almost same quantities of RbcL (55 kDa) (Fig 6C, lanes 1 and 4), were fractionated on sucrose gradients. Capsid proteins (42, 39 and 37 kDa) of purified wild-type viral particles were easily visualized after SDS-PAGE fractionation by Coomassie blue staining (Fig 6C, lane 2) and with anti-P4 antibodies (Fig 6C, panel 3), in contrast to purified CaMV-TAVm3 particles, for which larger aliquots were needed for their detection by staining (Fig 6C, lane 5) or immunodetection (Fig 6C, panel 6). Virus quantification indicated that plants infected with wild-type CaMV contained at least 50 times more particles than those infected with CaMV-TAVm3, *i*.*e*. 0.43 mg versus 0.008 mg of virions per gram of fresh turnip leaves.

**Fig 7:**
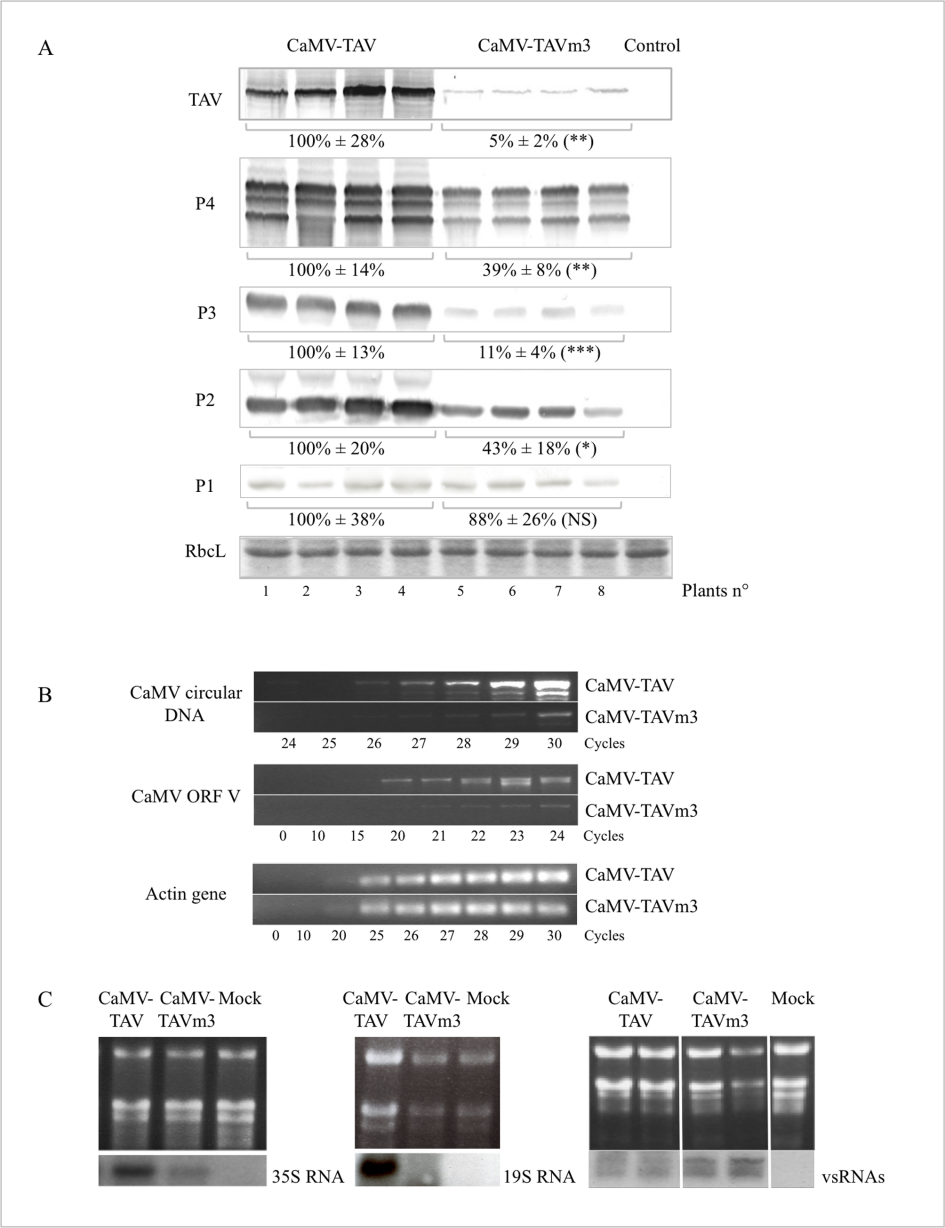
Analysis of CaMV proteins, DNA, 35S and 19S RNAs, and vsRNAs produced in turnip plants infected with wild-type CaMV or CaMV-TAVm3. (A) Immunodetection by western blot of CaMV’s TAV and TAVm3, P4 and the derived processed capsid proteins, P3, P2 and P1 proteins, using specific polyclonal antibodies and secondary antibodies coupled to alkaline phosphatase. Four independent infected plants were tested for CaMV-TAV (lanes 1–4) and CaMV-TAVm3 (lanes 5–8). Mock-inoculated plants were used as control. The loading control is RubisCO large subunit (RbcL) revealed by Coomassie blue staining. The bands corresponding to each of the 5 viral proteins were quantified as indicated in “Materials and Methods” and their corresponding values indicated, those of the viral proteins expressed in wild-type CaMV-infected plants being set at 100% (NS (not significant): p>0,5; * p<0.01; ** p<0.001; *** p<0.0001) (B) Semi-quantitative PCR performed on CaMV DNA from crude extracts of CaMV-TAV and CaMV-TAVm3-infected turnip plants using two couples of appropriate primers, which permit to only amplify a DNA sequence on the circular genome, and ORF V, respectively. The internal control was the actin gene *act2* amplified in the same crude extracts, by PCR using specific primers (S1 Table). The number of PCR cycles is indicated. (C) Northern blot performed on fractionated total RNA from plants infected by CaMV-TAV and CaMV-TAVm3, to detect the 35S and 19S RNAs (left and middle panels) and viral-derived small RNAs (vsRNAs) (right panel) using ^32^P-labeled oligonucleotides. RNAs from mock-inoculated plants were also probed.

The low number of CaMV-TAVm3 virus particles prompted us to compare the viral protein levels in plants infected with wild-type CaMV and CaMV-TAVm3 to determine whether their synthesis was affected in presence of TAVm3. Western blots were performed on total proteins extracted from leaf discs of infected turnip plants 21 dpi. The results were reproducible independently of the infection assay, as shown in Fig 7A. Overall, CaMV-TAVm3-infected turnip plants contained significantly lower amounts of viral proteins compared to plants infected by the wild-type virus (Fig 7A), and in particular TAVm3, whose quantity was estimated to represent only 5% of that of wild-type TAV. Treatment or not of seedlings of transgenic *Arabidopsis* expressing TAVm3 with the 26S proteasome inhibitor MG132 showed that the steady state level of TAVm3 remained unchanged throughout 24 hours, indicating that the low amounts of TAVm3 in CaMV-infected plants were not due to an instability of TAVm3 but rather to its reduced expression. The efficiency of the MG132 treatment was evidenced by the accumulation of the cellular protein RGA-DELLA in the presence of MG132, while this protein was barely detectable in mock-treated *Arabidopsis* seedlings (S5 Fig). Surprisingly, capsid proteins were present in plants infected with CaMV-TAVm3 at 39% of the levels of P4 detected in wild-type CaMV-infected plants, while we expected to detect minute amounts since CaMV-TAVm3 infected plants contained much less virus particles than wild-type CaMV-infected plants. We also noticed that the protein composition of the capsids (42, 37 and 35 kDa) was identical for CaMV and CaMV-TAVm3, indicating that the capsids were properly assembled and the pre-CP properly processed by the viral protease [42,43] in the cells infected by the mutant virus. For an unexplained reason, P3, another structural component of CaMV capsids, was found in very low amounts (11%) in CaMV-TAVm3-infected plants. The P2 protein was, on the contrary, produced in relatively high amounts (43%) in CaMV-TAVm3-infected turnip plants (Fig 7A), thus indicating that its inability to form ELIBs was due to events independent of its expression level. Finally, the movement protein P1 was expressed at almost similar levels (100% - 88%) in both CaMV- and CaMV-TAVm3-infected turnip plants partly explaining why the systemic propagation of infection occurred at the same rate for CaMV and CaMV-TAVm3.

These results suggest that the EKI mutation in TAV and/or EDIBs has a strong effect on the expression of TAVm3 protein itself but also of P3, and a rather weak or moderate effect on the expression of P1, P2 and P4, and on capsid assembly. However, despite the fact that the expression and processing of the capsid protein precursor remains almost unaffected, TAVm3 inability to form large EDIBs seems to strongly reduce the yield of CaMV progeny. Therefore, we favour the hypothesis that this poor yield in progeny is due to the instability of the CaMV particles.

### Replication of CaMV DNA by reverse transcription is strongly reduced in plants infected by CaMV-TAVm3 mutant

The discrepancy between the low yield of CaMV-TAVm3 progeny and the relatively high levels of capsid proteins in infected turnip plants strongly suggested that capsids might disassemble because they do not contain viral DNA. To verify this hypothesis, we performed semi-quantitative PCR that indicates DNA replication efficiency, on crude turnip extracts using a set of primers which anneal to the sequence that encodes the 5’ part of the 35S RNA leader region (forward primer), and to the ORF II encoding sequence (reverse primer), respectively, thus allowing the amplification of the circular viral genome [10] (S1 Table). PCR product analysis on ethidium bromide-stained agarose gel showed that three additional amplification cycles were required for the extract from CaMV-TAVm3-infected plants in order to obtain the same amounts of viral DNA than these amplified from extracts of wild-type CaMV-infected turnip plants (Fig 7B), while the actin gene *act2* used as an internal control was amplified following the same kinetic in both types of infected plants (Fig 7B). The use of another set of primers amplifying ORF V (S1 Table) led to the same conclusion (Fig 7B). Densitometry analyses, performed with the ImageJ software, indicate that plants infected with CaMV-TAVm3 only contained about 12% of the viral DNA amounts synthesized in turnip plants infected with wild-type CaMV, and thus, that viral DNA replication was strongly impaired in the presence of TAVm3.

Northern blots performed on total RNA using specific probes to detect CaMV transcripts showed that turnip plants infected with CaMV-TAVm3 contained approximately half the amount of 35S RNA compared to those infected with wild-type CaMV (Fig 7C, left panel). The 19S RNA coding for TAV was undetectable in CaMV-TAVm3-infected turnip plants, as opposed to those infected with CaMV-TAV (Fig 7C, middle panel). This suggests that the EKI mutation indirectly affected the transcription rate of the CaMV genome and/or the stability of viral RNAs. Analysis of virus-derived small RNAs (vsRNAs) revealed that plants infected with mutant CaMV contained slightly higher levels than in the context of an infection with the wild-type virus. Consequently, 35S and 19S RNAs were likely more susceptible to RNA silencing, despite the fact that the RNA silencing suppressor activity was not impaired in TAVm3 [12,44] (Fig 7C, right panel).

Taken together, our results confirmed that large electron-dense viroplasms are sites of intensive CaMV DNA replication by reverse transcription [45,46], where the 35S RNA is protected from degradation and capsid assembly is enhanced. However, in stark contrast with previous observations, our data suggest that large EDIBs are dispensable for the production of infectious CaMV particles, but we cannot rule out the possibility of the requirement for small TAV aggregates in the infection process.

## Discussion

The hallmark of CaMV infection is the formation of EDIBs [7], which are considered as virus factories. The size of these inclusion bodies is extremely variable and depends on the virus isolate, the largest being observed with the virulent Cabb B-JI isolate [32]. Previously, we showed that mutating the EKI sequence located at the N-terminus of the TAV protein (mutant TAVm3), the major component of EDIBs impaired this process in a non-viral context. In this paper, we describe the continuation of our investigation on EDIB formation and the analyses of their biological relevance for CaMV Cabb B-JI pathogenesis, by studying the TAVm3 CaMV mutant.

Testing the behaviour of TAV mutants in tobacco BY-2 cells revealed that three independent sequences of TAV are involved in the formation of inclusion bodies: the N-terminal EKI motif (amino acids 11–13), the C-terminal region of the MAV domain (amino acids 218–242) and a sequence encompassing the zinc finger (amino acids 413–462). Deletion of one of these sequences is sufficient to abolish the formation of large EDIBs, indicating that they participate collectively in this process. They roughly correspond to three of the four domains (D1, D2, D3 and D4) of the TAV protein of CaMV CM1841 isolate shown to interact with full-length TAV in yeast two-hybrid analyses: D1 (amino acids 1–110), D2 (amino acids 156–253) and D4 (amino acids 414–520) [47]. Mutation of the TAV EKI motif appeared to have a similar effect to deletion or point mutations of subdomain D3b (amino acids 309–343) [33] since both, Cabb B-JI TAVm3 and CM1841 TAV mutants, form small EDIBs in a non-viral context compared to the wild-type TAV proteins. Overlapping of domain D3b and RNA binding domain 2 (amino acids 330 to 379) [33] might partly explain the equivocal behaviour of CaMV Cabb-JI TAVΔRNA2 mutant in forming EDIBs or not in tobacco cells, depending on the assay.

In this study, we demonstrated that mutation of the EKI motif impairs the formation of large perinuclear EDIBs in turnip cells in a viral context, in Arabidopsis protoplasts expressing TAVm3 ectopically, and in protoplasts from TAVm3 transgenic Arabidopsis plants. Small TAVm3 inclusion bodies were formed in some cells, including CaMV-TAVm3-infected cells, whose formation is probably mediated by the two other interacting domains. This also suggests that the EKI mutation was not rescued by the upstream similar EKL tripeptide (Fig 1) in triggering TAVm3 self-assembly and the subsequent coalescence of small bodies to form large EDIBs.

The EKI sequence is conserved in all CaMV isolates (*i*.*e*. Cabb B-JI, D/H, D4, CM1841…) and several *Caulimoviruses*, such as *Dahlia mosaic virus* (DaMV) [48] and *Figwort mosaic virus* [49]. The TAV N-terminal region (domain A or D1) strongly interacts with the full-length protein *in vitro* [9] and *in vivo*, when tested in yeast two-hybrid assays [47]. This interaction is drastically reduced *in vitro* when EKI is mutated (data not shown), and *in vivo* when the glutamic acid and leucine at positions 312 and 316 of subdomain D3b are mutated in full-length TAV [33]. We believe that the EKI sequence interacts with subdomain D3b - it is conserved in many CaMV isolates, including Cabb B-JI—and that this potentiates the interactions between TAV molecules and/or TAV aggregates and, finally, the formation of large EDIBs. This hypothesis is supported by the observation that TAVm3 inclusion bodies never fused together to become large EDIBs, as is observed in wild-type CaMV-infected plants. Whether the EKI motif mediates the movement of TAV inclusion bodies along the cytoskeleton to reach the perinuclear region where the nucleation presumably occurs [2,26,50] remains an open question. Other experiments and a three-dimensional structure of the TAV protein are clearly needed to fully elucidate the mechanism leading to large EDIB formation in CaMV-infected cells, i.e. to determine whether sumoylation of TAV—two consensus sequences ΨKxD/E are present in TAV (M. Bureau, personal communication)–is required, as was shown to be the case for the generation of rotavirus viroplasms [51]. Deeper knowledge of this mechanism will certainly help to explain why EDIBs produced by some CaMV isolates remain small and/or do not fuse into larger viroplasms [32].

The CaMV Cabb B-JI isolate expressing TAVm3 systemically infects turnip plants but loses its virulence, as evidenced by the mild leaf symptoms (faint chlorosis) and the normal development of infected plants. Transgenic *Arabidopsis* lines (ecotype Columbia) encoding TAVm3 [12] also displayed a mild phenotype, resembling healthy plants, whereas lines expressing wild-type TAV showed severe leaf chlorosis and stunting, indicating that the EKI motif is an essential determinant in symptom expression and severity. Similarly to CaMV-TAVm3, CM1841 isolate harbouring mutations in the D3b domain also induced milder symptoms in turnip plants compared to those infected by the wild-type virus [33]. Studies performed with chimeric CaMV isolates, obtained by combining mild and severe CaMV isolates, have already demonstrated that the 5’ part of ORF VI, encoding TAV, is responsible for disease severity [16,52,53]. The domain responsible for symptomatology, at least in *Arabidopsis*, was precisely mapped to the distal region of domain D1 (amino acids 40–110) [44], thus excluding the EKI motif. In fact, since the C-terminal part of D1 is also involved in the RNA silencing suppression activity of TAV [44], this activity may contribute to the expression of symptoms in CaMV-infected plants by modifying the expression pattern of cellular genes as observed in transgenic *Arabidopsis* plants expressing TAV [54,55]. Concerning the N-terminal part of domain D1 (amino acids 1–20) that contains the EKI motif, it is required for the suppression of salicylic acid response gene expression [44]. Whether EKI plays a role in this process was not investigated in this study.

In summary, it appears that the EKI sequence is indirectly involved in symptom expression, probably by mediating the formation of large inclusion bodies, since none of TAV functions required for CaMV infectivity, including translation transactivation [11], nuclear import and export [12] and suppression of RNA silencing [44], is impaired when the EKI motif is mutated. Indeed, in turnip plants infected with the CaMV mutant, TAVm3 diffuses in the cytoplasm or forms few small aggregates, never triggering the formation of large EDIBs. Electron microscopy observations revealed that TAVm3 inclusion bodies are irregularly shaped and likely correspond to clusters of CaMV particles rather than EDIBs. This is in stark contrast with the sharp contour and uniform matrix of the small and large EDIBs contained in wild-type CaMV-infected turnip cells. Because viroplasms increase in size as synthesis of their components progresses [1,56], we thought that the inability of TAVm3 to self-assemble into typical EDIBs could be due to its low accumulation in infected turnip plants and/or to the host environment, as shown for the CaMV D4 isolate. In fact, the D4 wild-type TAV protein forms small EDIBs in turnip plants (1 μm^2^) but EDIBs dramatically increase in size (14 μm^2^) in *Datura*, the natural host of this isolate [32]. However, neither the abundance of TAVm3 nor the host context influence the behaviour of TAVm3, since large EDIBs resembling those found in turnip plants infected with wild-type CaMV were never observed in CaMV-TAVm3-infected *Arabidopsis* plants and in transgenic *Arabidopsis* lines, despite abundant synthesis of the TAV mutant [12].

A totally unexpected finding, made while analysing turnip mesophyll cells infected by CaMV-TAVm3 by electron microscopy, was the absence of electron-lucent inclusion bodies (ELIBs). Further studies using fluorescent microscopy revealed that the P2 protein, the major component of ELIBs, formed small aggregates scattered across the cytoplasm despite being present in high amounts, instead of forming a single large ELIB as in wild-type CaMV-infected cells, suggesting that TAV and/or EDIBs influence the behaviour of P2. The interaction between TAV and P2 [57] may induce a conformational change that triggers P2 self-assembly to form ELIB and/or allows its transport along the cytoskeleton network toward the nucleation site [50]. Consequently, the transmission of CaMV by aphids should be strongly affected in plants infected with CaMV-TAVm3, since neither EDIBs (the reservoirs of CaMV particles) nor ELIBs (the platforms for transmission) are formed in plants infected with the CaMV mutant [41,58].

The CaMV-TAVm3 mutant virus was able to systemically infect turnip plants, showing no delay compared to wild-type CaMV. Therefore, the mutation in TAVm3 did not perturb the cell-to-cell and the long distance movement of CaMV particles, in contrast with the mutation within domain D3 that affects TAV self-assembly and the rate of CaMV systemic infection [33]. A recent model proposed that TAV is involved in the intracellular movement of CaMV factories, since EDIBs were observed near plasmodesmata and TAV interacts with CaMV movement protein P1, PDLP1 (Plasmodesmatal-Located Protein 1) and AtSRC2 (Soybean Response to Cold) [25], two cellular proteins localized next to the tubules formed by P1 [30]. Decoration of the cytoskeleton with EGFP-TAVm3 in transient assays and the detection of small TAVm3 bodies along the plasma membrane of infected turnip protoplasts suggests that TAVm3 may still fulfil this hypothetic role.

Unexpectedly, CaMV-TAVm3 produced at least 50 times less virus particles than wild-type CaMV. However, infected turnip plants contained large amounts of processed capsid proteins, suggesting that this yield might be due to the scarce TAVm3 inclusions, since EDIBs are thought to be physical supports for capsid assembly [24]. Very recently, Hafrén *et al*. (2017) proposed that another function of EDIBs is to protect CaMV particles and capsid proteins against degradation by the NRB1-dependent autophagy mechanism [59]. Our data rather suggest that capsids were properly assembled, as the precursor P4 (pre-CP) is processed most likely after self-assembly [42,60]. CaMV proteins P1 and P2—P5 could not be tested in the absence of specific antibodies—were also produced at significant levels, indicating that the extremely low amount of TAVm3 was sufficient to efficiently activate reinitiation of 35S RNA translation. This also suggests that translation of CaMV transcripts occurred in the absence of typical EDIBs at alternative subcellular compartments, such as the endoplasmic reticulum or the microtubule network [21]. Consequently, we hypothesize that most TAV produced in the course of a wild-type CaMV infection accumulates to form EDIBs, while only a small soluble population of TAV is involved in translation reinitation and other functions such as suppression of RNA silencing. TAV, in its aggregated form, might act as a nucleation site for capsid assembly and encapsidation of viral DNA, and in particular as a chaperonin and/or scaffolding protein, since it physically interacts with P4 [24]. Champagne *et al*., (2004) found pre-CP and viral particles in small TAV bodies, while large EDIBs only contain mature CaMV particles. They proposed a model in which CaMV assembly starts soon after viral DNA synthesis, in small bodies in close proximity to the plasma membrane where pre-CP accumulates. After cleavage of the capsid precursor by the viral protease, the small bodies fuse to generate large EDIBs that consequently contain only mature CaMV particles [61]. The presence of CaMV particles in small TAVm3 bodies suggests that the latter are sites of virus assembly and processing of the pre-CP. However, the discrepancy between the amounts of processed capsid proteins and purified virus particles from CaMV-TAVm3-infected plants strongly suggested that most capsids were empty and/or disassembled in the absence of viral DNA and/or P3 which forms a network around the capsomers [62]. Our data clearly show that the level of CaMV DNA was drastically reduced in CaMV-TAVm3-infected turnip plants, since it represented only approximately 12% of the viral DNA level present in plant infected with wild-type CaMV. Processing of pre-CP by the viral protease indicates that precursor P5 was correctly cleaved *in cis* by the protease located at its N-terminus, thereby activating the reverse transcriptase [43]. Therefore, viral DNA synthesis in turnip plants infected with CaMV-TAVm3 was less active, most likely because large EDIBs, which are the physical supports for DNA replication complexes [45,46], are not formed in the presence of TAVm3. Reduced levels of viral DNA were also observed in turnip plants infected with a CaMV CM1841 isolate which codes for a TAV D3b mutant unable to trigger the formation of correctly sized EDIBs [33] but, by contrast to TAVm3, the subdomain D3b overlaps TAV region required for CaMV DNA synthesis [23]. The reduced DNA level may also be explained, at least partly, by the fact that CaMV-TAVm3-infected plants contain lower amounts of CaMV pregenomic 35S RNA, probably as a result of degradation, as evidenced by the detection of slightly higher amounts of vsRNAs compared to plants infected with wild-type CaMV [63,64]. As the RNA silencing suppressor activity of TAVm3 is unaffected [12] this suggests that the 35S and 19S RNAs were not physically protected from the RNA silencing machinery. Moreover, transcription of viral DNA yielded much less 35S and 19S RNAs since the nucleus was probably less re-infected by infectious particles and, consequently, a smaller amount of proteins was synthesized compared to an infection with wild-type CaMV.

In summary, we found that the mutation of the EKI motif within the N-terminal self-assembly domain of the TAV protein drastically affects the formation of proper EDIBs, and in particular of large EDIBs, and indirectly impacts the formation of CaMV transmission bodies (ELIB). This is harmful for CaMV DNA replication by reverse transcription and, subsequently, for an efficient production of infectious CaMV progeny, contributing to the loss of CaMV Cabb B-JI virulence on *Brassicaceae*. By contrast, in our experiments the EKI mutation did not hinder virus propagation within the plant, capsid morphogenesis or translation of viral 35S RNA, indicating that CaMV probably adapts by usurping subcellular compartments, as is the case for other *Caulimoviridae* members, such as *Petuviruses or Badnaviruses*, that do not express TAV homologues. Nevertheless, the functional contribution of the EKI motif in the establishment of an efficient CaMV infectious cycle certainly explains why it is conserved in the TAV protein of all CaMV isolates and in some *Caulimovirus* species.

## Supporting information

S1 Table **Oligonucleotides used as PCR primers to generate TAV-encoding cDNA and its derivatives (A) and for semi-quantitative RT-PCR (B).** PCR products encoding TAV and TAV mutants were cloned into pmRFP and pCK-EGFP vectors. The restriction sites, used for cloning, at the 5’ end of the primers are not indicated. Specific couples of primers were used for semi-quantitative RT-PCR to amplify a sequence of CaMV circular DNA and ORF V, and the *act-2* gene used as reference, respectively. Forward (+) and reverse (−) primers.
(TIF)

S1 Fig Enlargement of Fig 2B (panels 8–16) showing i) transient co-expression in BY-2 cells of: EGFP-A (A, panel 1), EGFP-TAVΔA (B, panel 1) or both (C, panel 1), and mRFP-TAV (A-C, panels 2); ii) DIC (differential interference contrast)-images (A-C, panels 3), and iii) merged images of panels 1–3 (A-C, panels 4). Scale bars: 10 μm.
(TIF)

S2 Fig Analysis of EGFP-TAV and EGFP-TAVm3 expression in *Arabidopsis* protoplasts. Protoplasts, prepared from 10 days-old *Arabidopsis* seedlings, were PEG400 (Sigma-Aldrich)-transfected with 15 μg carrier plasmid DNA and 10 μg of either pCK-EGFP-TAV or pCK-EGFP-TAVm3. Ectopically expressed EGFP-TAV and EGFP-TAVm3 were immunodetected by western blot 24h post-transfection in the whole protoplasts lysates with polyclonal rabbit antibodies against TAV (@-TAV) or EGFP, kindly provided by D. Gilmer (IBMP, Strasbourg, France) (@-EGFP), HRP (Horse Radish Peroxydase)-conjugated secondary goat anti-rabbit antibodies (Thermo Fischer Scientific), and luminol-based enhanced chemiluminescence substrate (Lumi-Light^Plus^ Western Blotting Substrate, Roche). The loading control (LC) is RubisCO large subunit (RbcL) revealed by Coomassie blue staining.
(TIF)

S3 Fig A Z-series stack can be played through the protoplast imaged in Fig 5A. The protoplast, observed by LSCM, was infected with wild-type CaMV and also transiently expressed EGFP-TAVm3. The P2 viral protein was immunodetected with specific anti-P2 antibodies and Alexa 568-conjugated secondary antibodies.
(AVI)

S4 Fig A Z-series stack can be played through the protoplast imaged in Fig 5B. The protoplast, observed by LSCM, was infected with CaMV-TAVm3 and also transiently expressed EGFP-TAVm3. The P2 viral protein was immunodetected with specific anti-P2 antibodies and Alexa 568-conjugated secondary antibodies.
(AVI)

S5 Fig Analysis of the stability of CaMV TAV protein in transgenic *Arabidopsis* seedlings. Eight-days-old transgenic *A*. *thaliana* seedlings expressing CaMV TAV [12] were incubated in MES buffer containing (+ MG132) or not (-MG132) 26S proteasome inhibitor MG132, for several hours at 21°C. At each time point (in hours), 10 seedlings were collected, ground, and proteins were analysed by western blot using anti-TAV and anti-RGA DELLA polyclonal antibodies and secondary antibodies coupled to alkaline phosphatase. Protein loading was controlled after transfer, by Ponceau S staining of the membrane (control). (-) corresponds to proteins in non-treated *Arabidopsis* seedlings before starting the proteasome inhibition experiment.
(TIF)
