# Supplementary figures and images for: Formation of large viroplasms and virulence of Cauliflower mosaic virus in turnip plants depend on the N-terminal EKI sequence of viral protein TAV
Source: PLoS One. 2017 Dec 18;12(12):e0189062. doi: 10.1371/journal.pone.0189062 (PMC5734791; doi:10.1371/journal.pone.0189062)

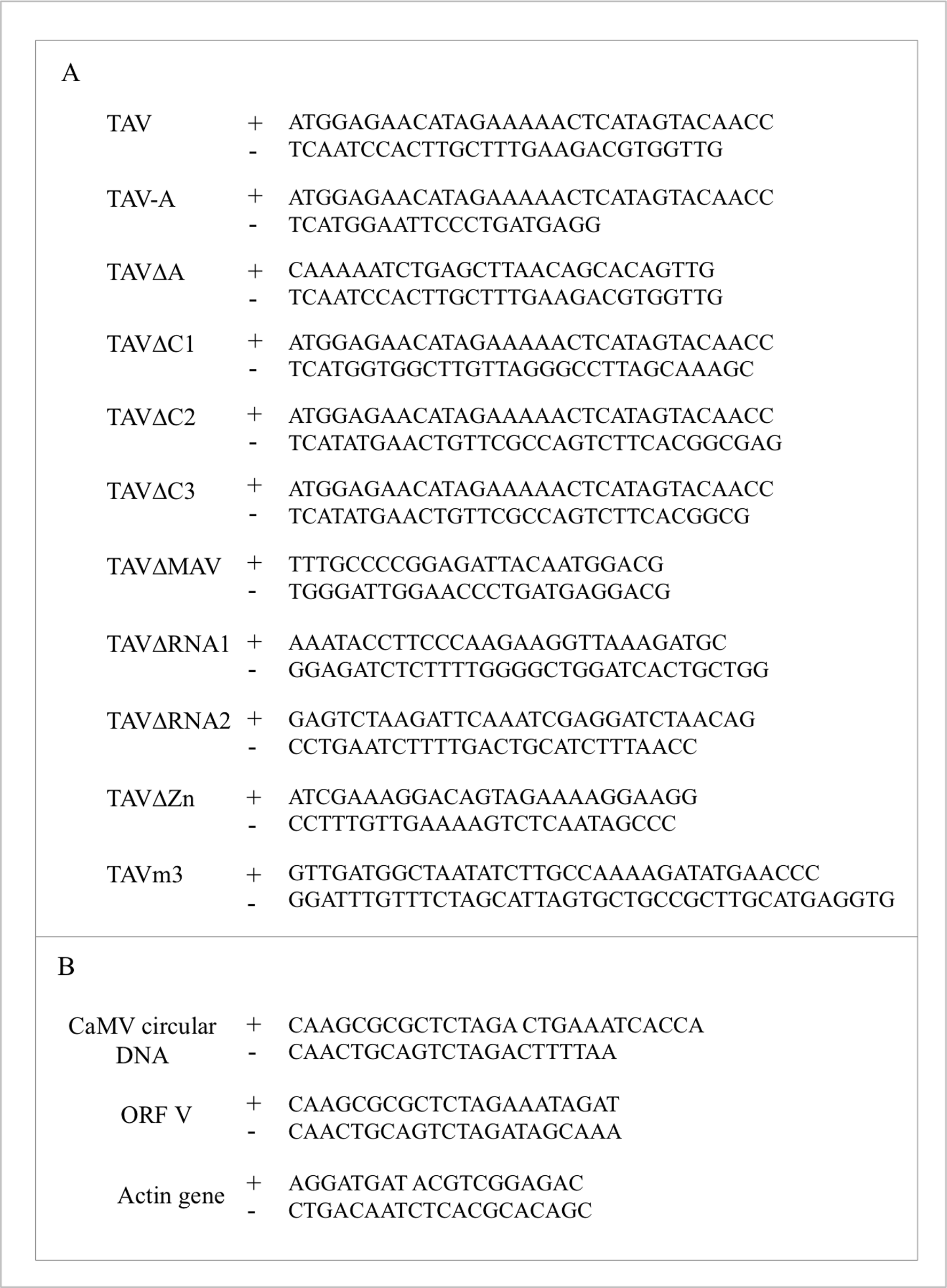

Supplement: S1 Table — Oligonucleotides used as PCR primers to generate TAV-encoding cDNA and its derivatives (A) and for semi-quantitative RT-PCR (B). PCR products encoding TAV and TAV mutants were cloned into pmRFP and pCK-EGFP vectors. The restriction sites, used for cloning, at the 5’ end of the primers are not indicated. Specific couples of primers were used for semi-quantitative RT-PCR to amplify a sequence of CaMV circular DNA and ORF V, and the act-2 gene used as reference, respectively. Forward (+) and reverse (−) primers. (TIF) [file pone.0189062.s001.tif]

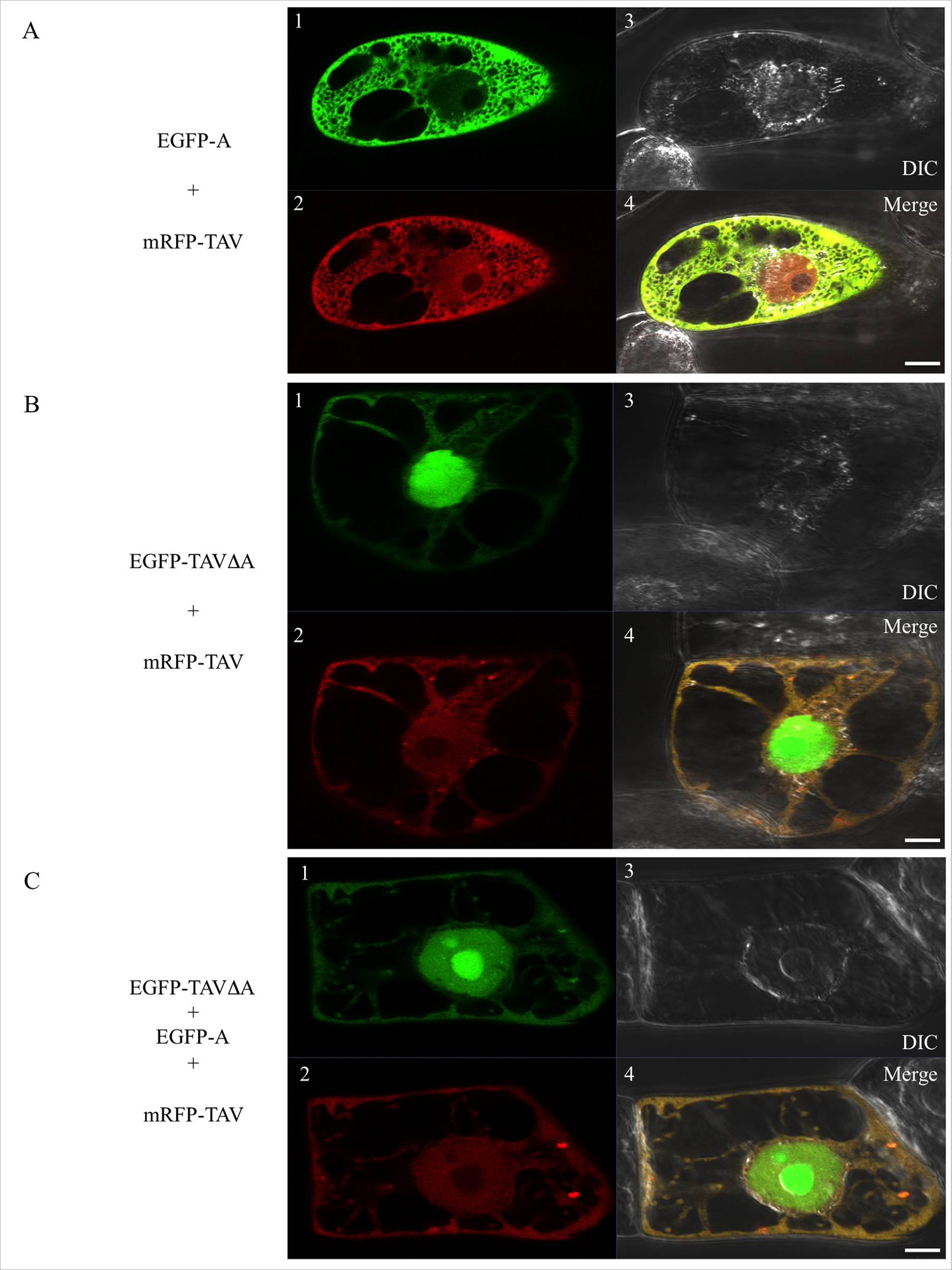

Supplement: S1 Fig — Enlargement of Fig 2B (panels 8–16) showing i) transient co-expression in BY-2 cells of: EGFP-A (A, panel 1), EGFP-TAVΔA (B, panel 1) or both (C, panel 1), and mRFP-TAV (A-C, panels 2); ii) DIC (differential interference contrast)-images (A-C, panels 3), and iii) merged images of panels 1–3 (A-C, panels 4). Scale bars: 10 μm. (TIF) [file pone.0189062.s002.tif]

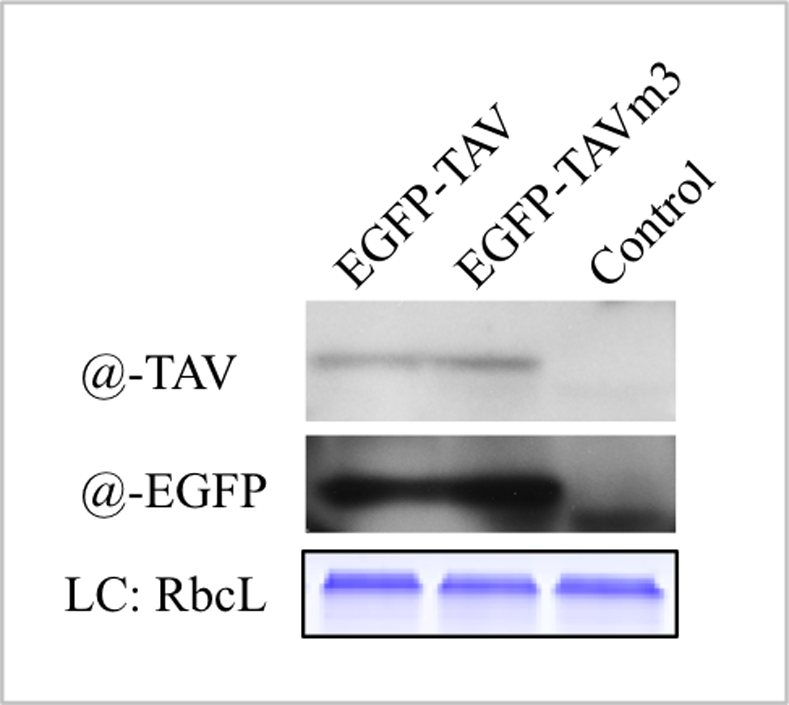

Supplement: S2 Fig — Protoplasts, prepared from 10 days-old Arabidopsis seedlings, were PEG400 (Sigma-Aldrich)-transfected with 15 μg carrier plasmid DNA and 10 μg of either pCK-EGFP-TAV or pCK-EGFP-TAVm3. Ectopically expressed EGFP-TAV and EGFP-TAVm3 were immunodetected by western blot 24h post-transfection in the whole protoplasts lysates with polyclonal rabbit antibodies against TAV (@-TAV) or EGFP, kindly provided by D. Gilmer (IBMP, Strasbourg, France) (@-EGFP), HRP (Horse Radish Peroxydase)-conjugated secondary goat anti-rabbit antibodies (Thermo Fischer Scientific), and luminol-based enhanced chemiluminescence substrate (Lumi-LightPlus Western Blotting Substrate, Roche). The loading control (LC) is RubisCO large subunit (RbcL) revealed by Coomassie blue staining. (TIF) [file pone.0189062.s003.tif]

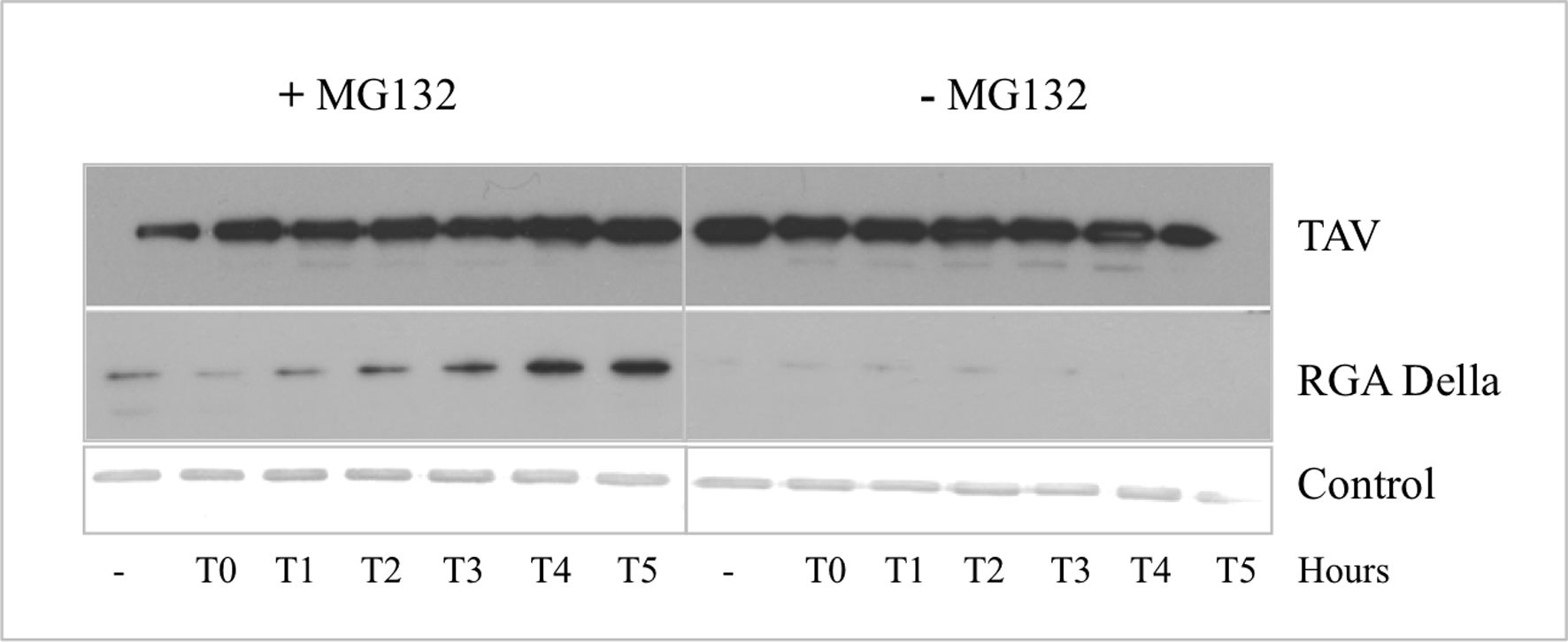

Supplement: S5 Fig — Eight-days-old transgenic A. thaliana seedlings expressing CaMV TAV [12] were incubated in MES buffer containing (+ MG132) or not (-MG132) 26S proteasome inhibitor MG132, for several hours at 21°C. At each time point (in hours), 10 seedlings were collected, ground, and proteins were analysed by western blot using anti-TAV and anti-RGA DELLA polyclonal antibodies and secondary antibodies coupled to alkaline phosphatase. Protein loading was controlled after transfer, by Ponceau S staining of the membrane (control). (-) corresponds to proteins in non-treated Arabidopsis seedlings before starting the proteasome inhibition experiment. (TIF) [file pone.0189062.s006.tif]
